# TAC3 Gene Products Regulate Brain and Digestive System Gene Expression in the Spotted Sea Bass (*Lateolabrax maculatus*)

**DOI:** 10.3389/fendo.2019.00556

**Published:** 2019-08-14

**Authors:** Zhanxiong Zhang, Haishen Wen, Yun Li, Qing Li, Wenjuan Li, Yangyang Zhou, Lingyu Wang, Yang Liu, Likang Lyu, Xin Qi

**Affiliations:** Key Laboratory of Mariculture, Ministry of Education, Ocean University of China, Qingdao, China

**Keywords:** spotted sea bass, TAC3/TACR3 system, neurokinin B, *in situ* hybridization, growth

## Abstract

Neurokinin B (NKB) is a member of the *tachykinin* (*tac*) family that plays important roles in mammalian growth by modulating prolactin (PRL) synthesis and secretion and causing contraction of the stomach and intestine. However, its potential role in regulating growth of teleosts is less clear. We aimed to explore the role that NKB plays in regulating fish growth using the spotted sea bass (*Lateolabrax maculatus*) as a model. In the present study, two *tac3* and two *tacr3* genes were identified in the spotted sea bass. Sequence analysis showed that two *tac3* transcripts, *tac3a* and *tac3b*, encode four NKBs: NKBa-13, NKBa-10, NKBb-13, and NKBb-10. Expression analysis in different tissues showed that both genes are highly expressed in the brain, stomach and intestine of the spotted sea bass. *In situ* hybridization indicated that the *tac3a* and *tac3b* mRNAs are both localized in several brain regions, such as the telencephalon and hypothalamus, and that *tacr3a* and *tacr3b* are localized in the intestinal villus and gastric gland. To investigate the potential role of NKBs in regulating growth, *in vitro* experiments were performed to detect the effect of NKBs on growth-related gene expression in the brain and brain-gut peptide (BGP)-related genes in the stomach and intestine. NKBb-13 was the most critical ligand in regulating the expression of growth-related genes in the brain and brain-gut peptide (BGP)-related genes in the stomach. The expression of *cholecystokinin* (*cck*) was enhanced by NKBa-13, NKBa-10, and NKBb-10 but not NKBb-13 in the intestine. In general, our results showed that NKBs participate in regulating the growth of spotted sea bass.

## Introduction

Neurokinin B (NKB), which is encoded by *tachykinin 3* (*tac3*), is a member of the tachykinin family (1, 2). NKBs are peptides conserved from invertebrates to mammals with C-terminal motifs of FVGLM-NH2 (3). NKB has been studied in several teleost groups and has multiple functions, including the control of smooth muscle contraction in the gastrointestinal tract (2, 4), vasodilation to modulate blood pressure (5), fluid secretion in the gut epithelium (6), and even the regulation of sperm motility (7). The biological actions of NKB are mediated mainly by NK3 receptor, which is a member of the rhodopsin-type class I group of G protein-coupled receptors (2, 8, 9).

In recent years, studies on NKB were focused on its role in regulating reproduction in many species. Gonadotropin-releasing hormone (GnRH) was believed to be the primary factor that controls the synthesis and release of luteinizing hormone (LH) and follicle-stimulating hormone (FSH) from the pituitary gonadotrophs, which then guide the gonad development (10). Kisspeptins and their cognate receptor, GPR54, regulate the secretion of GnRH (11). The loss of function mutations in the genes encoding TAC3 or TACR3, whose translation products are NKB and its receptor, lead to hypogonadotrophic hypogonadism in humans (10, 12). NKB was found to be able to generate GnRH pulsatility and regulate downstream LH release *via* NK3 receptor within the hypothalamus in rodents and sheep (2, 13, 14). In teleost, NKB was found to regulate reproduction by multiple pathways, including negatively modulating kisspeptin2 to regulate the release of GnRH1 in the striped bass (15), stimulating the release and synthesis of LH in zebrafish, tilapia and goldfish (10, 16–18). These findings suggested that NKB also play an important role in regulating fish reproduction.

In the rat, NKB caused contraction of the intestine and NK3 receptor expression in the small intestine and stomach, including the pylorus (19). In rat intestine, both receptors for tachykinins and the tachykinin signaling pathway were characterized in dispersed muscle cells (20). The brain and gut form the brain-gut axis through bidirectional nervous, endocrine, and immune communications (21). Gastrointestinal motility is regulated through the brain-gut axis, which consists of two sides: one is the nerve conduction while the other is the endocrine regulation. The central nervous system (CNS) plays an important role in regulating gut function and homeostasis. After integrating various pieces of information from various centers of the brain and spinal cord when they receive signals regarding internal or external environmental changes, the CNS transmits its regulatory information to the enteric nervous system (ENS) or directly acts on gastrointestinal effector cells through the autonomic nervous system and the neuroendocrine system to regulate the smooth muscle and glands (22). There is also endocrine regulation in gastrointestinal motility except nerve conduction, which is mainly achieved by brain-gut peptides. Brain-gut peptide is not only the material basis of the brain-gut pathway, but also an important target in the brain-gut axis (23). Tachykinins are important excitatory neurotransmitters in the enteric nervous system involved in the coordination of gastrointestinal motility (24, 25). Based on recent *in vitro* studies in grass carp, NKBs were found to be novel stimulators of prolactin (PRL) and somatolactin α (SLα) secretion and gene expression *via* the activation of NK3 receptor expressed in the carp pituitary (2). It is noteworthy that NKBs stimulate the release of growth hormone (2). Tachykinins were detected in the nerves of the gastrointestinal tract, where they can cause the contraction of the esophageal smooth muscle (26). Meanwhile, the molecular mechanisms behind are less clear. In this study, we employed the spotted sea bass to investigate the role of NKBs in fish growth. Previous studies have indicated that NKBs are important neuroendocrine regulators of growth-associated gene expression in teleosts and gastrointestinal motility in mammals, and the mechanisms involved in these functions are worth discussing.

NKB was found to upregulate PRL and somatolactin (SL) secretion and transcript expression in the pituitary (27). SL, a member of the growth hormone (GH/PRL) family, is released from the neurointermediate lobe (NIL) of the posterior pituitary (28, 29). A study in grass carp suggested that *tac3* gene products stimulate PRL and SLα synthesis and secretion through the activation of different NK3 receptor subtypes coupled to overlapping postreceptor signaling mechanisms (27). However, functional research on the *tac3/tacr3* system in teleost growth has only focused on the pituitary (27).

In this study, the cDNAs coding *tac3s* and *tacr3s* were cloned and were localized in the brain by *in situ* hybridization, stomach and intestine. To evaluate their functions in regulating growth, the mRNA levels of *motilin (mln), ghrelin (ghrl), cholecystokinin (cck)*, and *gastrin (gas)* were measured after the NKB stimulation of *in vitro* cultured stomach and intestinal fragments and the mRNA levels of *growth hormone-releasing hormone (ghrh), prolactin-releasing hormone (prlh), insulin-like growth factor-1 (igf1)*, and *gh* were measured after NKB stimulation in cultured brain cells.

## Materials and Methods

### Animals and Chemicals

Male spotted sea bass, with body weight (bw) of 0.7–0.8 kg, were obtained from a local fish market in Qingdao, China. All animal experiments were approved and performed in accordance with the respective Animal Research and Ethics Committees of Ocean University of China. The fish were anesthetized with tricaine methanesulfonate (MS-222) (30). Tissue samples were collected immediately and frozen in liquid nitrogen.

### RNA Extraction and cDNA Preparation

Total RNA was extracted from spotted sea bass (*n* = 3) tissues (telencephalon, midbrain, hypothalamus, cerebellum, medulla oblongata, pituitary, liver, stomach, intestine, spleen, gill, heart, muscle, kidney, head kidney, and testis) using TRIzol reagent (Invitrogen). The quantity and quality of the RNA were estimated using a biophotometer (OSTC, China) and agarose gel electrophoresis, respectively. The RNA was reverse-transcribed to complementary DNA (cDNA) using 1 μg of total RNA per 20 μL reaction with a Prime Script^TM^ RT reagent kit (TaKaRa, Japan) according to the manufacturer's instructions.

### Sequence Analysis of *tac3* and *tacr3*

According to the genome (unpublished), the open reading frames (ORFs) of *tac3a, tac3b, tacr3a, tacr3b* in the spotted sea bass, *Lateolabrax maculatus*, were predicted, and gene-specific primers were designed. To amplify cDNA fragments of the spotted sea bass *tac3* and *tacr3* genes, PCR was performed using specific primers corresponding to spotted sea bass NKB cDNA sequence. All primers used in this study are listed in Table 1.

**Table 1:** Primers used in the present study.

**Gene**	**Forward primer (5'-3')**	**Reverse primer (3'-5')**	**AT**
**PRIMERS FOR FULL-LENGTH PCR**			
tac3a	ATGAGGAGAGGATTGCTGTTG	CTACAGCCCCTGAAGAAACC	56
tac3b	ATGGAGAGAACTCCAAACTG	CTACAGAAAGCGTTGAAACC	56
tacr3a	ATGGCGGCTCCACACAAC	TCAAGAGAACTCCTCAGGTT	56
tacr3b	ATGGCCTCTGAACGGGAT	CTAACTGCCTTTATTATTGG	55
**PRIMERS FOR REAL-TIME PCR**			
tac3a	GAGGAGAGGATTGCTGTTGGTGAC	TCCAAGCCTACGGTCGGATCTG	61
tac3b	TTGCTGCGGAACACAAGGATGG	AACCTCTGCCTGCCATGATTGATG	60
tacr3a	AACCTCTGCCTGCCATGATTGATG	CCGTGTGGCGAGATGTGATGTAG	62
tacr3b	CAAGCGGATGCGGACTGTCAC	CTAACTGCCTTTATTATTGG	61
Motilin (mln)	TGCTGATGAAGGAGCGAGAA	TCCACCATGTTCCACCTGAG	56
Ghrelin (ghrl)	ACACCTGTTTGCTGGTCTTTC	ATGTGATGTGGTTGGCCTCTG	56
Gastrin (gas)	TGCTAAGAGGGAGAAACTG	TATCTCGCGTTCATCGTC	55
Cholecystokinin (cck)	TGCCAACTACAACCAACCT	GCGTCGTCCAAAGTCCAT	56
ghrh	GGCCGGAGGGATACTTCCAT	GCAGAGATTTGGCCCAGGAC	59
prlh	TGCTCCTCCTCCTCCTCTCCTC	CACGCTGCTGTGCCTCTTCC	62
gh	AAGAGTGGTCCTCCTGCTGTCC	TTGTTGAGTTGACGCTGCTCCTC	61
igf1	CATTGTGGACGAGTGCTGCTTCC	CTTGTCTGGCTGCTGTGCTGT	61
18S	GGGTCCGAAGCGTTTACT	TCACCTCTAGCGGCACAA	56

The PCR amplification protocol was performed as our previous report (10): initial denaturation at 94°C for 3 min followed by 30 cycles at 94°C for 30 s, 55–60°C for 30 s and 72°C for 1 min. The reaction was terminated with further extension for 5 min at 72°C. The amplification products were purified using a TIANgel Midi Purification Kit (TIANGEN, China) and subcloned into the pEASY-T1 cloning vector (TransGen Biotech, China). Positive clones containing inserts of the expected size were selected for sequencing to confirm their sequence.

The signal peptide and neuropeptide prohormone cleavage sites in *tac3s* were predicted using SignalP 4.1 (31) and NeuroPred software (32, 33), respectively. Putative seven-transmembrane domains were predicted using the TMHMM server v. 2.0 (http://www.cbs.dtu.dk/services/TMHMM-2.0/) (34). Multiple sequences were aligned and compared using Clustal X (35), and a phylogenetic tree was constructed by MEGA 6 using the neighbor-joining method (33, 36).

### Tissue Expression Analysis

For the tissue distribution analysis, three fish were sampled in each group. The mRNA levels of *tac3* in different tissues (telencephalon, midbrain, hypothalamus, cerebellum, medulla oblongata, pituitary, gill, liver, intestine, stomach, spleen, heart, kidney, head kidney, muscle, and testis) were evaluated by real-time PCR, which was performed in triplicates as previously described (10). In detail, real-time PCR was performed with a Step One Plus Real-Time PCR System using a SYBR Green I Kit (TaKaRa, Japan) according to the manufacturer's instructions. The expression of spotted sea bass *tac3a, tac3b, tacr3a*, and *tacr3b* was detected by primers that generated a unique fragment with a size of 117, 92, 199, and 192 bp, respectively. After initial denaturation at 95°C for 30 s, each template was amplified with 40 cycles of denaturation for 5 s at 95°C and annealing for 30 s at 60°C. The concentration of the template in the sample was determined by relating the Ct value to a standard curve. The *tac3a, tac3b, tacr3a*, and *tacr3b* transcript levels were normalized against 18S transcript levels. Primers used to amplify *tac3a, tac3b, tacr3a*, and *tacr3b* are listed in Table 1.

### *In situ* Hybridization (ISH)

The *in situ* hybridization was performed as we previously reported (17, 37). The brains, stomachs, and intestines of male spotted sea bass were removed and fixed in buffered 4% paraformaldehyde for 24 h and then embedded in paraffin. Seven-micron-thick sections were cut for ISH. The sections were pasted onto aminopropylsilane-treated glass slides and dried in an oven at 37°C. Sense and antisense digoxigenin (DIG)-labeled riboprobes about 200–300 bp in length were synthesized from the open reading frames (ORFs) of the spotted sea bass *tac3s* and *tacr3s* genes using a DIG RNA Labeling Kit (Roche Diagnostics, Mannheim, Germany). The sections were briefly rehydrated by a graded series of ethanol solutions (100–80%) after being cleared in xylene, permeabilized with 0.1 M HCl for 10 min followed by proteinase K (10 μg/ml) digestion for 20 min, washed in 1×PBS for 10 min, then washed in 2×SSC for 10 min, prehybridized at 55°C for 1 h, and hybridized with DIG-labeled riboprobes diluted in hybridization buffer (17) at 55°C overnight in a wet box. To obtain the best results for ISH, the concentrations of probes were tested before the final experiment. The concentrations were 50 μg/ml for tac3s in telencephalon and tac3a in midbrain and hypothalamus, and the concentrations were 500 μg/ml for tacr3s in stomach and intestine and tac3b in midbrain and hypothalamus. The type of salt and concentration in the hybridization buffer were as follows: 50% deionized formamide, 25% 20×SSC, 10% 50×Denharts, 1 mg/ml yeast tRNA, 5% Water-DEPC, 100 mg/ml dextran sulfate. After hybridization, the sections were washed in grade series of SSC, PBS Solution and blocked with blocking reagent (Roche Diagnostics). DIG was detected with an alkaline phosphatase-conjugated anti-DIG antibody (Roche Diagnostics; diluted 1:3,000), and chromogenic development was conducted with an NBT/BCIP stock solution (Roche Diagnostics) (17).

### Brain Cell Culture and *In vitro* Stomach and Intestine Incubation Assays

Peptides corresponding to spotted sea bass NKBs (NKBa-13, NKBa-10, NKBb-13, and NKBb-10) were synthesized by GL Biochem in Shanghai, China. Their purity was >95% as determined by analytical HPLC. The NKBa-13, NKBa-10, NKBb-13, and NKBb-10 activities were further examined in the primary cells of spotted sea bass brains and the tissues of spotted sea bass stomachs and intestines.

The cell and tissue culture were performed according our previous report with slight modifications (37). In brief, five spotted sea bass, weighting 0.7–0.8 kg, were anesthetized with MS-222 before decapitation. The isolated brains were collected, washed three times with phosphate-buffered saline (PBS) and then cut into small pieces under sterile conditions. The fragments were digested with 25 mg/10 ml trypsin at 25°C for 20 min, and 20% fetal bovine serum (FBS) was added to terminate protease digestion (10). Brain cells were harvested by centrifugation (1,200 rpm for 5 min) and cultured in a 24-well plate containing 1 mL medium 199 (M199) with 100 U/mL penicillin, 100 mg/mL streptomycin, and 20% fetal bovine serum (FBS) (37). After preincubation at 28°C for 2 h, the medium was aspirated, and fresh culture medium was added. After 3–4 h, the medium was changed into fresh culture medium containing NKBs (10^−7^, 10^−6^ M) or medium alone was added. The cells were harvested after incubation for 3 h and 6 h and stored at −80°C for subsequent RNA extraction and real-time PCR (37). The experiments were performed in triplicate in three independent experiments. The mRNA levels of *ghrh, prlh, gh, igf1* were determined in both treatment group and control group. The 2^−ΔΔCT^ method was used to determine the relative mRNA levels in the different groups. The mRNA level in the control was considered to be 1, and the expression in treatment cells was calculated as a fold of the expression level in controls.

The stomachs and intestines from five fish were collected and washed three times with phosphate-buffered saline (PBS), cut into small pieces (1 mm^3^), and cultured in a 24-well plate containing 1 mL medium 199 (M199) with 100 U/mL penicillin, 100 mg/mL streptomycin, and 20% fetal bovine serum (FBS) (37). After preincubation at 28°C for 2 h, the medium was changed into fresh culture medium containing NKBs (10?7, 10?6 M) or medium alone was added. The stomach and intestinal fragments were harvested after incubation for 3 and 6 h and stored at −80°C for subsequent RNA extraction and real-time PCR. The experiments were performed in triplicate in three independent experiments. The mRNA levels of *gas, mln*, and *ghrl* were determined in the stomachs treatment group and control group, and the mRNA levels of *cck, gas, mln*, and *ghrl* were also examined in treatment group and control group. The 2^−ΔΔCT^ method was used to determine the relative mRNA levels in the different groups. The mRNA level in the control was considered to be 1, and the expression in treatment cells was calculated as a fold of the expression level in controls. The experiments were triplicated to further confirm the result.

### Statistical Analysis

All data were expressed as the mean values ± S.E.M. Date analyses were performed by one-way ANOVA followed by Duncan's multiple range test, and significance was considered at *P* < 0.05. All statistics were performed using SPSS 19.0 (SPSS, Chicago, IL, USA).

## Results

### Identification and Synteny Analysis of the *tac3* and *tacr3* Genes in the Spotted Sea Bass

**Figure 1:**
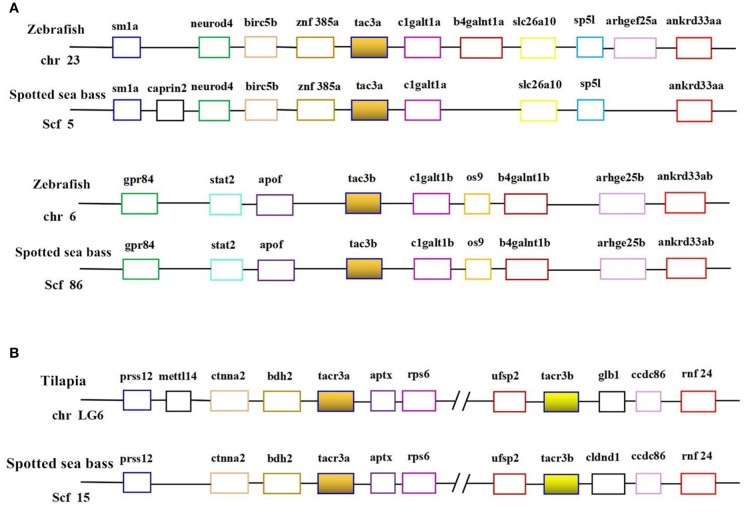
Syntenic analysis of *tac3s* **(A)** in zebrafish and spotted sea bass and *tacr3s* **(B)** in tilapia and spotted sea bass. In this map, the spotted sea bass genes are immediately proximal to each other on the chromosome, and the zebrafish *tac3s* genes and tilapia *tacr3s* genes are located in the same chromosomal region.

Genomic data mining suggested that two *tac3* and two *tacr3* genes were present in the spotted sea bass genome. The evolutionary relationship between the *tac3* genes in zebrafish and spotted sea bass and the relationship between *tacr3* genes in tilapia and spotted sea bass were established, and we performed genomic synteny analysis (Figures 1A,B). Orthologous relationships were identified between spotted sea bass *tac3a/tac3b* and zebrafish *tac3a/tac3b* and spotted sea bass *tacr3a/tacr3b* and tilapia *tacr3a/tacr3b*. Synteny was clearly observed for *tac3a* and *tac3b*, which were closely linked to *birc5b, znf385a*, and *clgaltla* in zebrafish and *apof* and *clgalt1b* in spotted sea bass. Similarly, *ctnna2* and *bdh2* were upstream of *tacr3a*, and *aptx* was located downstream of *tacr3a* in both tilapia and spotted sea bass. We also found that *ufsp2, ccdc86*, and *rnf24* were linked to *tac3b* in tilapia and spotted sea bass. Accordingly, syntenic analysis showed that *tac3s* and *tacr3s* were relatively conserved in evolution.

**Figure 2:**
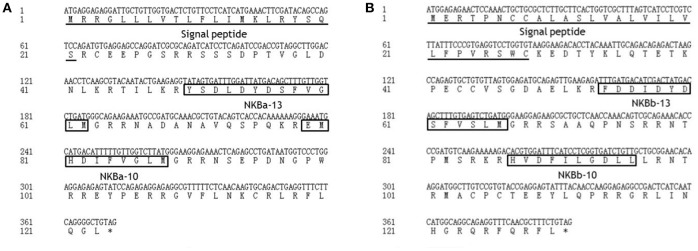
Nucleotide and deduced amino acid sequences of spotted sea bass *tac3a* **(A)** and *tac3b* **(B)**. The putative signal peptides are underlined. The putative NKB peptides are boxed.

**Figure 3:**
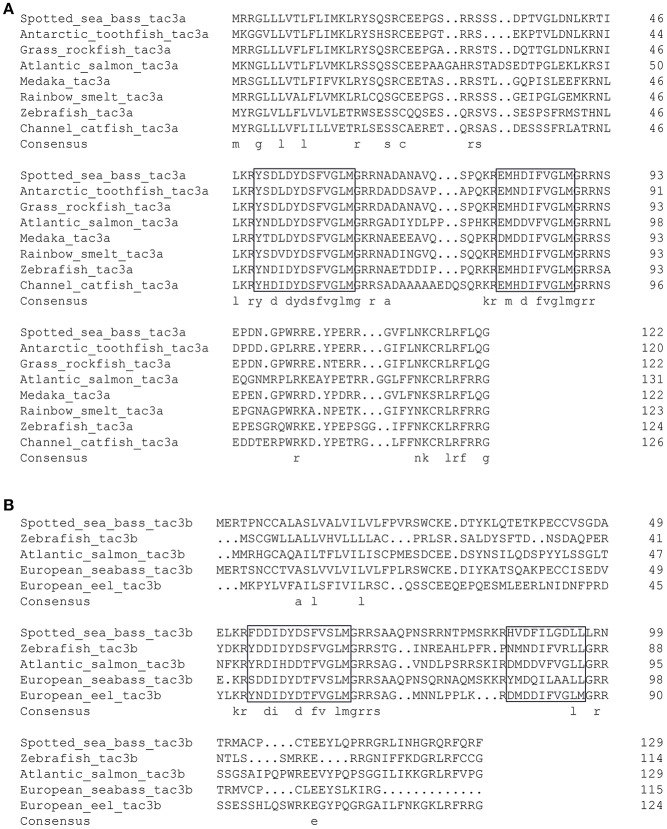
Comparison of the amino acid sequences of spotted sea bass *tac3a* **(A)** and *tac3b* **(B)** precursors from different species. The boxed letters indicate the sequence of mature NKB peptides in the detected species.

As shown in Figure 2, the ORF of *tac3a* is 372 bp, coding a 124 amino acid (aa) precursor with a predicted signal peptide of 21 aa (Figure 2A). The ORF of *tac3b* is 393 bp, coding a 131 aa precursor with a predicted signal peptide of 28 aa (Figure 2B). Sequence analysis showed that each precursor contains two putative peptides. The four spotted sea bass tachykinin peptides were designated NKBa-13, NKBa-10, NKBb-13, and NKBb-10 according to their length. Sequence analysis showed that NKBa-13 and NKBa-10 share a common C-terminal sequence (FVGLM). The C-terminal of NKBb-13 and NKBb-10 was a modification of that motif, with -FVSLM-NH2 at the C-terminus and -LGDLL-NH2 at the C-terminus, respectively. Sequence alignment between teleostean *tac3* precursors revealed that NKBa-10 and NKBa-13 are well conserved, but NKBb-10 and NKBb-13 exhibited low similarity in teleost fish (Figures 3A,B).

**Figure 4:**
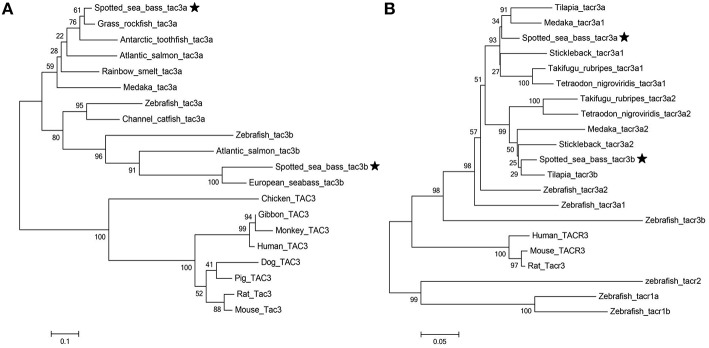
Phylogenetic analysis of the *tac* **(A)** and *tacr* **(B)** gene family in vertebrates. The phylogenetic tree was constructed by MEGA 6 using the neighbor-joining method. Data were resampled with 1,000 bootstrap replicates.

Phylogenetic trees of the *tac3s/tacr3s* gene families were constructed. As shown in Figure 4A, piscine *tac3s* and *tac3s* from other vertebrates formed two large clades, and the piscine clade was subdivided into *tac3a* and *tac3b* clades, with each clade containing spotted sea bass *tac3a* and *tac3b*, respectively. As shown in Figure 4B, spotted sea bass *tacr3a* was more closely related to tilapia *tacr3a* and medaka *tacr3a1* than to the other *tacr3* sequences, and spotted sea bass *tacr3b* was more closely related to tilapia *tacr3b* than to the other peptides.

### Tissue Expression of Spotted Sea Bass *tac3s/tacr3s*

**Figure 5:**
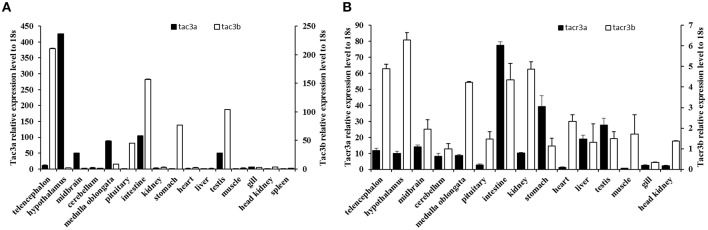
Expression of *tac3s* **(A)** and *tacr3s* **(B)** mRNAs in various tissues (telencephalon, midbrain, hypothalamus, cerebellum, medulla oblongata, pituitary, gill, liver, intestine, stomach, spleen, heart, kidney, head kidney, muscle, and testis) of male spotted sea bass. The X axis indicates different tissues. The results are normalized to 18S RNA. The data are shown as the mean ± S.E.M (*n* = 3) and the mRNA levels of *tac3a* and *tac3b* were calculated as fold change relative to the mRNA levels of the heart and spleen, respectively, while the mRNA levels of *tacr3a* and *tacr3b* were calculated as fold change relative to the mRNA levels of the heart and pituitary, respectively.

The expression patterns of the *tac3* and *tacr3* genes are shown in Figure 5. *Tac3a* mRNA was mainly expressed in the hypothalamus, with low levels expressed in the medulla oblongata, midbrain, testis, and intestine (Figure 5A). However, it was not detected in the muscle, liver, or kidney. *Tac3b* was highly expressed in the telencephalon, pituitary, stomach, intestine, and testis. Both *tacr3s* were widely expressed in all the tissues examined (Figure 5B). *Tacr3a* was highly expressed in the intestine, stomach and testis, and the highest expression of *tacr3b* was observed in the hypothalamus and intestine.

### Localization of the mRNA of *tac3s* in the Brain and the mRNA of *tacr3s* in the Stomach and Intestine

**Figure 6:**
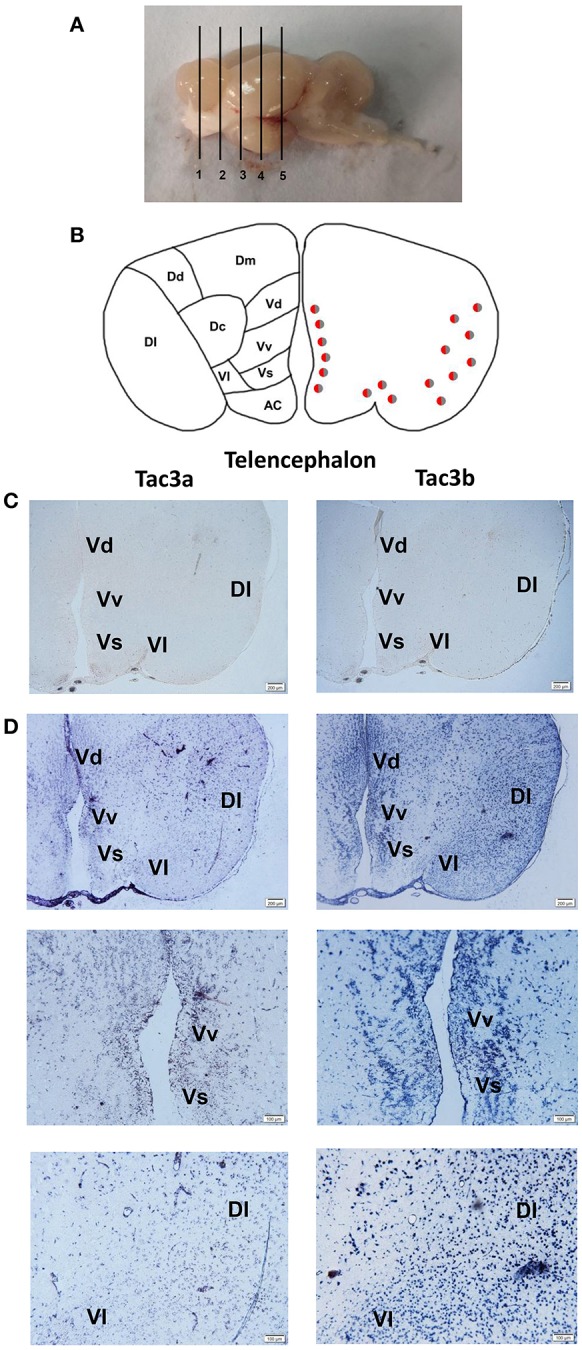
Photomicrographs and schematic illustrations of the mRNA levels of *tac3s* in the brain of the spotted sea bass, as detected by *in situ* hybridization (ISH). Schematic illustrations showing the positions of the slides within the brain **(A)**. Schematic drawings of spotted sea bass telencephalon **(B)**. Photomicrographs of *tac3a* and *tac3b* sense probe results in the telencephalon **(C)**. Positive signal of *tac3a* and *tac3b* in the telencephalon **(D)**. VM, ventromedial thalamic nucleus; Vv, area ventralis telencephali pars ventralis; Vs, area ventralis telencephali pars supracommissuralis; Vd, Area ventralis telencephali pars dorsalis; Dl, area dorsalis telencephali pars lateralis; Vl, area ventralis telencephali pars lateralis; Dm, area dorsalis telencephali pars medialis; Dc, area dorsalis telencephali pars centralis; Dd, area dorsalis telencephali pars dorsalis; AC, anterior commissure; Vd, area ventralis telencephali pars dorsalis. Red and gray dots represent the locations of *tac3a* and *tac3b*, respectively.

**Figure 7:**
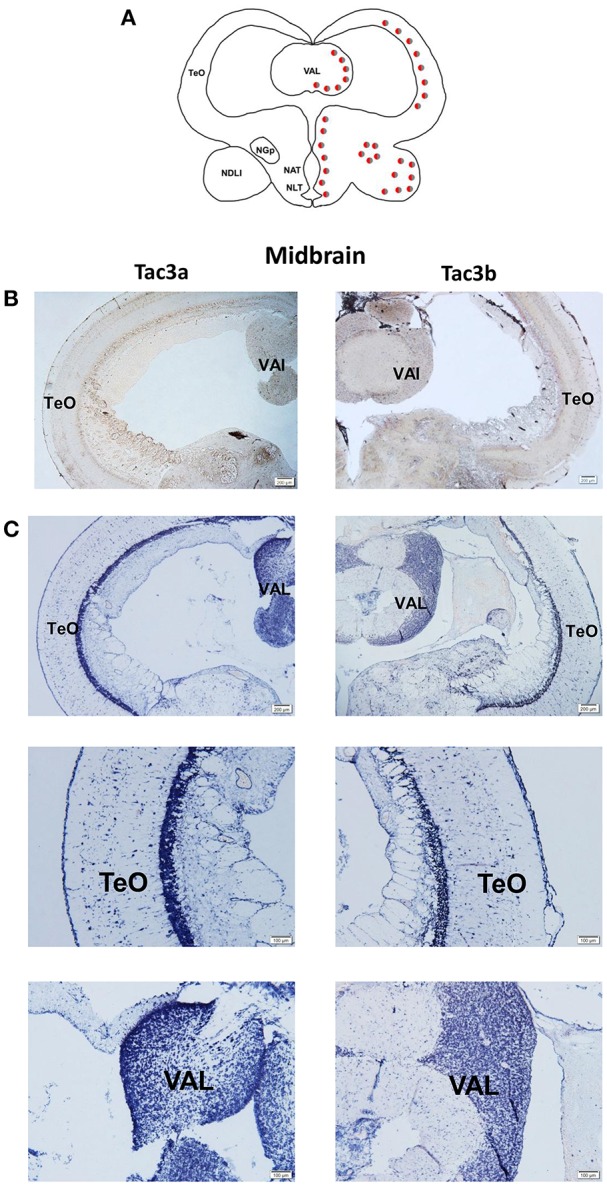
Photomicrographs and schematic illustrations of the mRNA levels of *tac3s* in the midbrain of the spotted sea bass, as detected by *in situ* hybridization (ISH). Schematic drawings of spotted sea bass midbrain **(A)**. Photomicrographs of *tac3a* and *tac3b* sense probe results in the midbrain **(B)**. Positive signal of *tac3a* and *tac3b* in the midbrain **(C)**. Abbreviations: TeO, tectum opticum; Val, lateral division of the valvula cerebella. Red and gray dots represent the locations of *tac3a* and *tac3b*, respectively.

**Figure 8:**
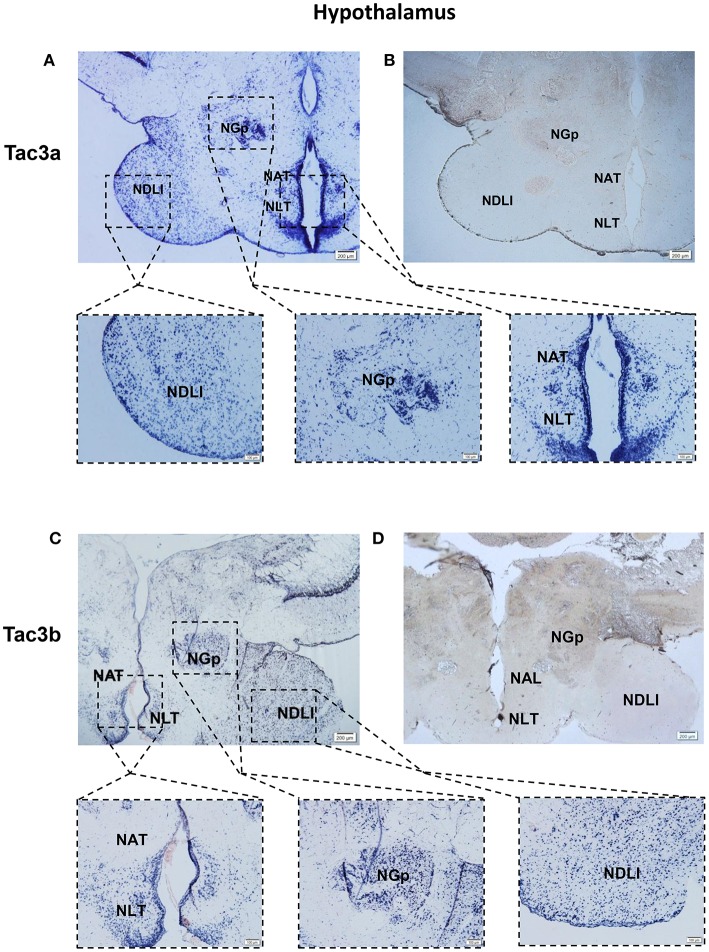
Photomicrographs and schematic illustrations of the mRNA levels of *tac3s* in the hypothalamus of the spotted sea bass, as detected by *in situ* hybridization (ISH). Photomicrographs of *tac3a* and *tac3b* sense probe results in the hypothalamus **(B,D)**. Positive signal of *tac3a* and *tac3b* in the hypothalamus **(A,C)**. NDLl, diffuse nucleus of the inferior lobe; NLT, lateral tuberal nucleus; NAT, Nucleus anterior tuberis; NGp, posterior nucleus glomerulosus; TeO, tectum opticum; Val, lateral division of the valvula cerebella; Red and gray dots represent the locations of *tac3a* and *tac3b*, respectively.

The *tac3a* and *tac3b* mRNA distribution in the brain was determined by ISH. Tissue sections were prepared from the brain, stomach, and intestine of spotted sea bass, and ISH was performed. The *tac3s* positive signals were detected in some brain regions, and mainly located in several areas. Localization of both *tac3a* and *tac3b* in the brain is shown in Figures 6–**8**. Strong signals for *tac3a* and *tac3b* mRNA were observed in several areas of the telencephalon (Figure 6D). *Tac3a* and *tac3b* signals were also detected in the lateral division of the valvula cerebellum (Val) (Figure 7C), the tectum opticum (TeO) (Figure 7C), the posterior part of the nucleus glomerulosus (NGp) (Figures 8A,C), the nucleus anterior tuberis (NAT) (Figures 8A,C), the nucleus lateralis tuberis (NLT) (Figures 8A,C), and the diffuse nucleus of the inferior lobe (NDLI) (Figures 8A,C). In addition, *tac3a* and *tac3b* signals seem to be detected in other type of cells such as glial cells (Figure 7C).

**Figure 9:**
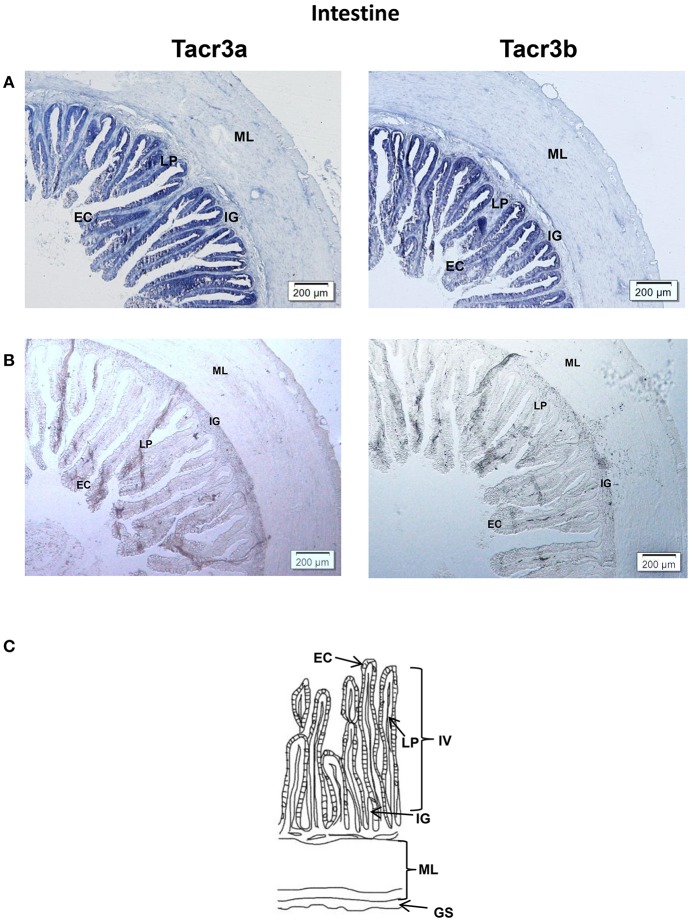
Photomicrographs and schematic illustrations of the mRNA levels of *tacr3s* in the intestine of the spotted sea bass, as detected by *in situ* hybridization (ISH). Positive signal of *tacr3a* and *tacr3b* in the intestine **(A)**. Photomicrographs of *tacr3a* and *tacr3b* sense probe results in the intestine **(B)**. Schematic diagram for intestine histology **(C)**. GS, gastric serosa; ML, muscular layer; IG, intestinal gland; IV, intestinal villus; LP, lamina propria; EC, epithelial cell.

**Figure 10:**
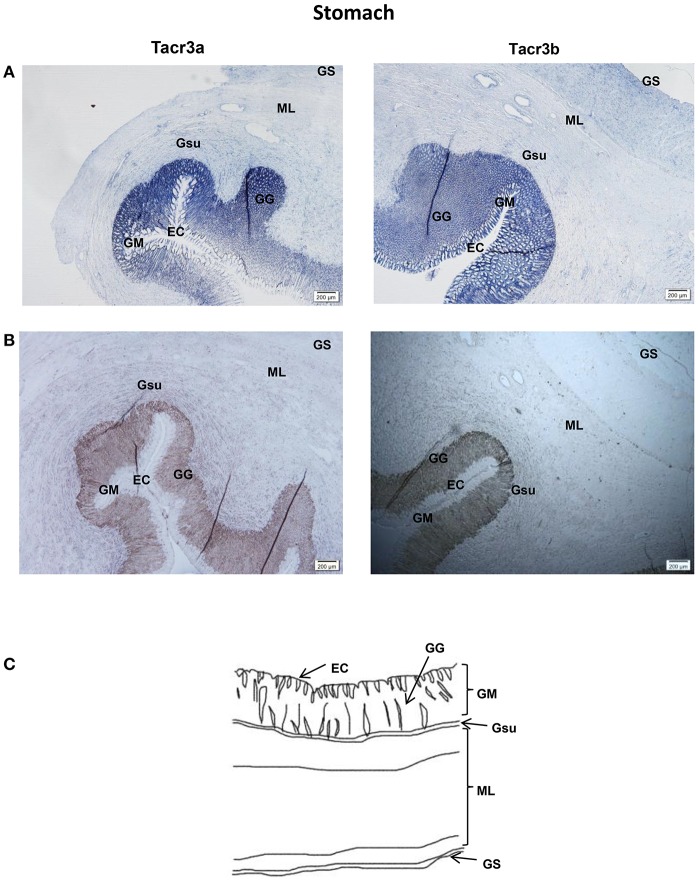
Photomicrographs and schematic illustrations of the mRNA levels of *tacr3s* in the stomach of the spotted sea bass, as detected by *in situ* hybridization (ISH). Positive signal of *tacr3a* and *tacr3b* in the stomach **(A)**. Photomicrographs of *tacr3a* and *tacr3b* sense probe results in the stomach **(B)**. Schematic diagram for stomach histology **(C)**. ML, muscular layer; EC, epithelial cell; GM, gastric mucosa; Gsu, gastric submucosa; GS, gastric serosa; GG, gastric gland.

*Tacr3a* and *tacr3b* mRNA localization in the stomach and intestine was also determined by ISH. Schematic diagram of stomach and intestine are showed in Figures 9C, 10C. The spotted sea bass intestine consists of the intestinal villus (IV), the intestinal gland (IG), and the muscular layer (ML). The intestinal villus (IV) is a leaf-like structure composed of the epithelial cell (EC) (LP) and the lamina propria facing the intestinal lumen (37). Strong signals for *tacr3a* and *tacr3b* mRNA were found in both the epithelial cell (EC) and the lamina propria (LP), while no expression was observed in the muscular layer (ML) or intestinal gland (IG) (Figure 9A). The spotted sea bass stomach consists of the gastric mucosa (GM), the gastric submucosa (Gsu), the muscular layer (ML), and the gastric serosa (GS). The gastric mucosa (GM) formed by epithelial cell (EC) and the gastric gland (GG) faces the gastric lumen. Strong signals for both *tacr3a* and *tacr3b* mRNA were observed in the gastric gland (GG) and epithelial cell (EC), while no signal was detected in the gastric submucosa (Gsu), the muscular layer (ML) or the gastric serosa (GS) (Figure 10A). No signal was detected using the sense probe in Figures 9B, 10B.

### The Effect of NKBs on the mRNA Levels of Growth-Related Genes in the Brain *In vitro*

To evaluate the effect of NKBa-13, NKBa-10, NKBb-13, and NKBb-10 on the expression of growth-related genes in spotted sea bass, *in vitro* studies to determine the effect of NKBs on the mRNA levels of *ghrh, prlh, gh*, and *igf1* were performed in primary cultured brain cells. The effect of NKBs on the mRNA levels of growth-related genes *in vitro* are listed in the Table 2. The *ghrh, prlh, gh*, and *igf1* sequences were identified in the spotted sea bass genome. The target genes were amplified and sequenced to confirm the correction of the mRNA sequence for real-time PCR.

**Table 2:** The effect of NKBs on the mRNA levels of growth-related genes and brain-gut peptide (BGP)-related genes *in vitro*.

**Region**		**NKBa-13**	**NKBa-10**	**NKBb-13**	**NKBb-10**
Brain	Ghrh	–	–	+	+
	Prlr	+	+	+	–
	Gh	–	–	–	–
	Igf1	–	+	–	–
Stomach	gas	+	–	+	–
	mln	–	–	+	–
	ghrl	–	–	+	–
Intestine	cck	+	+	+	–
	gas	–	–	–	+
	mln	–	+	–	–
	ghrl	–	–	–	–

“*+”, NKBs are effective after treatment; “–”, NKBs have no effect after treatment*.

**Figure 11:**
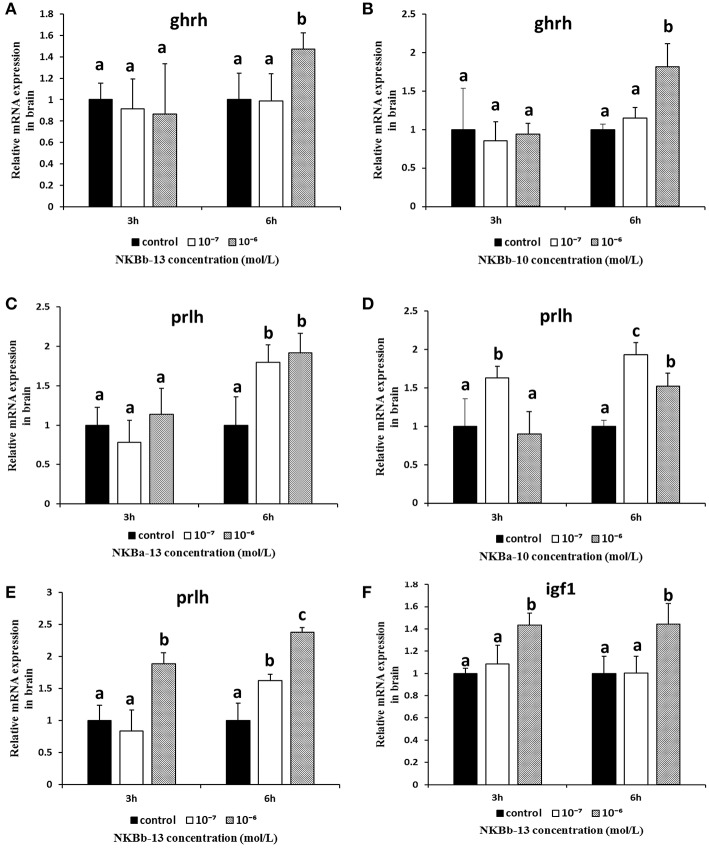
Effect of NKBs on the expression of growth-related genes in the brain. The X axis indicates hours after NKB treatment. **(A,B)** Effect of NKBb-13 and NKBb-10 on the mRNA levels of *ghrh* in the brain. **(C–E)** Effect of NKBa-13, NKBa-10, and NKBb-13 on the mRNA levels of *prlh* in the brain. **(F)** Effect of NKBb-13 on the mRNA levels of *igf1* in the brain. The results were represented as the mean ± SEM and expressed as fold of the expression level in controls. Significant differences are noted by different letters for each concentration (*P* < 0.05).

As shown in Figure 11, NKBb-13 and NKBb-10 treatment alone both significantly elevated *ghrh* mRNA levels in brain cells after treatment for 6 h, and the most significant enhancement was seen at a concentration of 10^−6^ M (Figures 11A,B). NKBa-13 and NKBa-10 had no significant effect on *ghrh* mRNA levels. As shown in Figures 11C–E, NKBa-10, NKBa-13, and NKBb-13 significantly increased the mRNA levels of *prlh* in brain cells 6 h after treatment. NKBa-13 and NKBb-13 at a concentration of 10^−6^ M had a significant effect on *prlh* mRNA levels, but NKBa-10 at a concentration of 10^−7^ M upregulated *prlh* mRNA levels. Only NKBb-13 significantly increased *igf1* mRNA levels, but no significant difference was observed between 3 and 6 h after treatment (Figure 11F). In contrast, the four NKBs had no effect on the expression of *gh* in the brain cells. Negative results of NKBs on the mRNA levels of related growth-related genes were showed in Supplementary Figure 1.

### The Effect of NKBs on the mRNA Levels of Brain-Gut Peptide (BGP)-Related Genes in the Stomach and Intestine *In vitro*

To further evaluate the effect of NKBa-13, NKBa-10, NKBb-13, and NKBb-10 on spotted sea bass, *in vitro* studies of the effect of NKBs on the mRNA levels of *gas, mln*, and *ghrl* were performed in intestine fragments. The effect of NKBs on the mRNA levels of brain-gut peptide (BGP)-related genes *in vitro* is listed in Table 2. The *cck, gas, ghrl*, and *mln* sequences were identified in the spotted sea bass genome.

**Figure 12:**
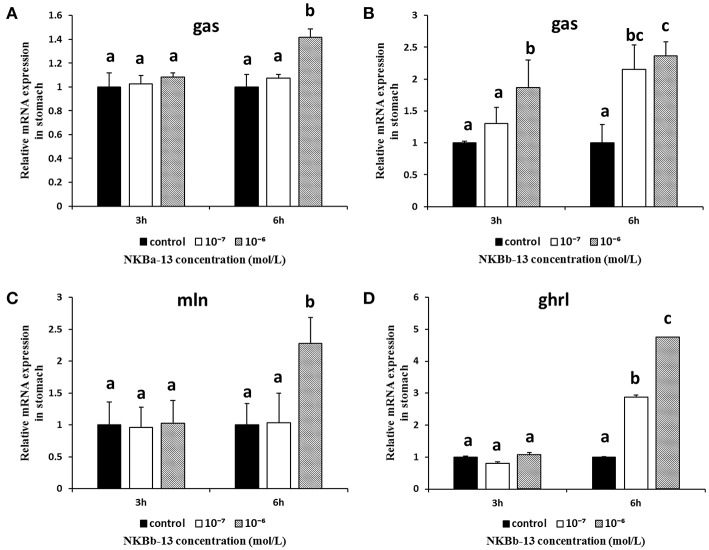
Effect of NKBs on the expression of brain-gut peptide (BGP)-related genes in the stomach. The X axis indicates hours after NKB treatment. **(A,B)** Effect of NKBa-13 and NKBb-13 on the mRNA levels of *gas* in the stomach; **(C,D)** Effect of NKBb-13 on the mRNA levels of *gas* and *ghrl* in the stomach; the results are represented as the mean ± SEM and expressed as fold of the expression level in controls. Significant differences are noted by different letters for each concentration (*P* **<** 0.05).

As shown in Figures 12A,B, NKBa-13, and NKBb-13 significantly increased the mRNA levels of *gas* in the stomach 6 h after treatment, and the two peptides both had a significant effect at a concentration of 10^−6^ M. As shown in Figures 12C,D, the mRNA levels of *mln* and *ghrl* were significantly increased after treatment with NKBb-13 for 6 h, and their levels were highest with an NKBb-13 concentration of 10^−6^ M. The other NKBs had no obvious effect on the mRNA levels of the three brain-gut peptide (BGP)-related genes.

**Figure 13:**
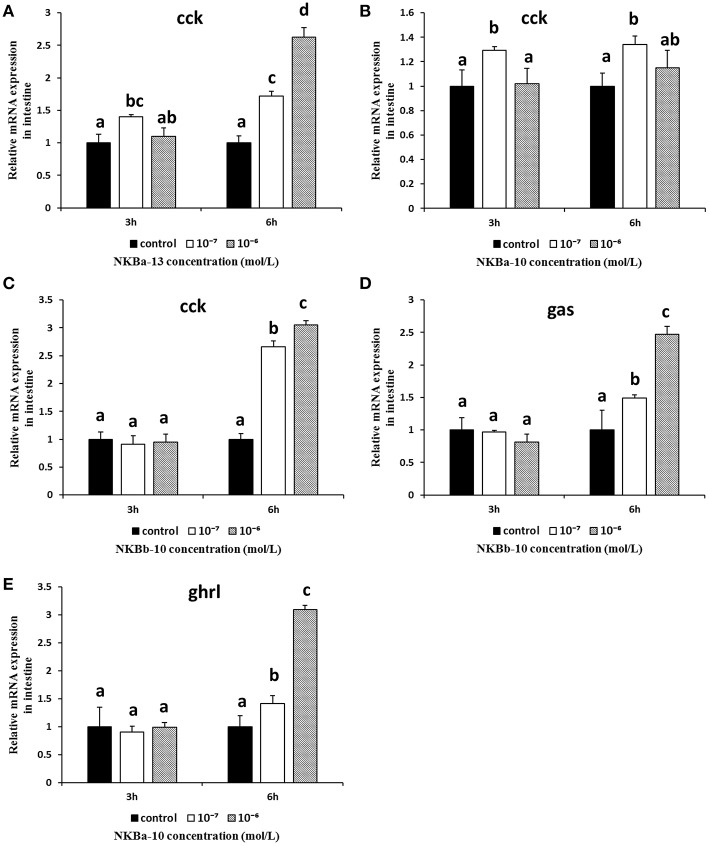
Effect of NKBs on the expression of brain-gut peptide (BGP)-related genes in the intestine. **(A–C)** Effect of NKBa-13, NKBa-10, and NKBb-10 on the mRNA levels of *cck* in the intestine. The X axis indicates hours after NKB treatment. **(D)** Effect of NKBb-10 on the mRNA levels of *gas* in the intestine. **(E)** Effect of NKBa-10 on the mRNA levels of *ghrl* in the intestine. The results are represented as the mean ± SEM and expressed as fold of the expression level in controls. Significant differences are noted by different letters for each concentration (*P* < 0.05).

The *in vitro* effects of NKBs on the mRNA levels of *cck, gas, mln*, and *ghrl* were also examined in intestine fragments. As shown in Figures 13A–C, the mRNA levels of *cck* were obviously increased after NKBa-13 and NKBb-10 treatment for 6 h, especially at a concentration 10^−6^ M. NKBa-10 at a concentration of 10^−7^ M significantly increased the mRNA levels of *cck*, but it had no significant effect after 3 and 6 h. *Gas* mRNA was also significantly increased following NKBb-10 treatment with a concentration of 10^−6^ M than compared to concentrations of 10^−7^ M and in the control group (*P* < 0.05), but there was no significant difference between treatment with different concentrations of NKBb-10 after incubation for 3 h (Figure 13D). The other three NKBs had no obvious effect on the expression of *cck* mRNA. NKBa-10 significantly stimulated the expression of *ghrl* mRNA after treatment for 6 h, and the most significant enhancement was seen with a concentration of 10^−6^ M (Figure 13E). These results suggested that neither NKBa nor NKBb significantly enhanced the mRNA levels of *mln* in incubated intestinal fragments. Negative results of NKBs on the mRNA levels of related brain-gut peptide (BGP) genes were showed in Supplementary Figures 2, 3.

## Discussion

An increasing number of studies have reported that the NKB/NK3 receptor system plays a critical role in teleosts (10, 17, 34); however, studies on NKBs in teleosts are limited to reproduction. In the present study, to identify the effects of NKBs on growth in teleost fish, we amplified and characterized two spotted sea bass *tac3* cDNAs and determined their mRNA levels' patterns and regulation in the brain, stomach, and intestine. Our findings suggested that NKBs as neuroendocrine regulators of growth in fish.

Previous studies reported two *tac3* genes (*tac3a* and *tac3b*) in zebrafish, goldfish and salmon (10, 16, 34). In the present study, we also identified two *tac3* and two *tacr3* genes in the spotted sea bass genome. There were two tachykinin peptides in the spotted sea bass *tac3* precursor, which were conserved in fish species, suggesting common functions in teleosts. The two spotted sea bass *tac3* genes generate four mature NKB peptides. The common C-terminal FVGLM-NH2 motif of spotted sea bass NKBa-13 and NKBa-10 is known to be important for its bioactivity (12, 17). Spotted sea bass NKBb-10 contains LGDLL-NH2. A substitution in an amino acid in the NKBb-13 C-terminus (from FVGLM-NH2 to –FVGLT-NH2) has been associated with changes in bioactivity. Therefore, NKBb-13 may have different bioactivities than other NKBs. However, more suitable approaches, advanced methods and further studies are still needed.

Q-PCR results showed that *tac3a* and *tac3b* were much more strongly expressed in the brain, consistent with findings in other fish (10, 16, 17, 27, 34, 38). *tac3a* and *tac3b* were also expressed at high levels in the stomach, intestine, and testis. These results are different from previous results in goldfish that less *tac3a* expressed in the intestine, but similar to those in the orange spotted grouper (10, 17). Previous results also showed that zebrafish *tac3* mRNAs were widely expressed in the central nervous system and peripheral tissues (34). These results suggest that *tac3* expression varies among species (17).

*Tac3* signals were observed in several brain regions using *in situ* hybridization. The areas in the brain in which *tac3* were expressed in the spotted sea bass, such as the telencephalon, lateral division of the valvula cerebella, tectum opticum and the nucleus anterior tuberis, were similar to those observed in the orange-spotted grouper (10). Similar to the goldfish, *tac3s* mRNA was found in some brain regions where no signal was detected—neither in zebrafish nor in orange-spotted grouper—including the diffuse nucleus of the inferior lobe and nucleus anterior tuberis. However, in the preoptic region, no signal for *tac3* were observed, which was different from both zebrafish and goldfish. The different expression pattern may be due to interspecific difference or differences in physiological state. In addition, *tac3s* signals were also found in several glial cells. It has been well established that NKB is a critical modulator of GnRH regulating the mammalian puberty onset and reproductive physiology (10, 13) and is involved in the regulation of the reproductive axis in some fish species. Although the *tac3* expressed differently in several brain regions of different species, the highest *tac3* expression is rather conserved in the brain, strongly suggesting its involvement in the regulation of growth in fish. In the intestine, both receptors were highly expressed in the epithelial cells (ECs) of the intestinal villi (IV) and lamina propria (LP), while they were highly expressed in epithelial cells (ECs) and the gastric gland (GG) in the stomach, implying that *tacr3s* may regulate the secretion of digestive juices or gastrointestinal motility-associated peptides. It has been shown that NK3 receptor is present on enteric neurons (6, 39–41). Tachykinins cause contraction of the esophageal smooth muscle, and NK3 receptor may mediate the motility-stimulating action of different tachykinins in the intestine (26, 42). Tachykinins act as neurotransmitters on neurons and cells (such as the secretory epithelium, smooth muscle cells, and glands) in the gastrointestinal tract of mammals (24). They are important excitatory neurotransmitters in the enteric nervous system involved in the coordination of gastrointestinal motility (24, 26). The distribution of *tacr3* mRNA in the stomach intestine was related to previous research, and it may be a potential explanation of gastrointestinal activities mediated through NK3 receptor. The expression of both ligands and receptors in stomach, intestine, and neuronal tissues suggested the physiological significance of this system in spotted sea bass growth.

To further study the function of NKBs in the brain, we detected growth-related gene (*ghrh, prlh, gh*, and *igf1*) expression after NKB treatment. The physiological effects of NKBb-13 and NKBb-10 increased the expression of *ghrh* after 6 h of treatment, while they had no effect on the expression of *gh*. *Gh* is mainly expressed in the pituitary. The stimulation of *gh* expression by NKBb-13 and NKBb-10 may be mediated indirectly *via* the *ghrh* in the hypothalamus of the spotted sea bass. A recent study in grass carp demonstrated that NKBs stimulated prolactin and somatolactin a synthesis and secretion from the pituitary (17, 27). NKBa-13, NKBa-10, and NKBb-13 significantly increased the expression of *prlh* mRNA in brain cells. NKB induced PRL release in rat pituitary cells (43). NK3 receptor may mediate the expression of PRLH in the spotted sea bass brain. Only NKBb-13 significantly increased *igf1* mRNA levels. The present study has revealed that NKBb-13 is the most critical ligand among the NKB peptides in regulating the expression of growth-related genes in the spotted sea bass brain.

*Ghrl, mln, gas*, and *cck* are brain-gut peptides with the ability to stimulate prokinetic activity on gastrointestinal motility (37, 44). In this study, the effect of NKBs on the mRNA levels of gastrointestinal-related genes (*ghrl, gas*, and *mln*) in the stomach fragments was detected. We found that only NKBb-13 significantly increased the mRNA levels of *gas, ghrl*, and *mln* in the stomach, which may explain a previous study, in which tachykinins acted as neurotransmitters in the gastrointestinal tracts of mammals (24). We also investigated the effect of NKBs on mRNA levels of gastrointestinal-related genes (*ghrl, gas, cck*, and *mln*) in the intestinal fragments. The present study showed that all NKB peptides, except NKBb-13, significantly up-regulated the mRNA levels of *cck*. Only NKBb-10 upregulated the mRNA levels of *gas*, and only NKBa-10 significantly increased the mRNA levels of *ghrl*. The increased expression of *cck* mRNA is a potential factor in spotted sea bass intestinal motility that may be mediated by NK3 receptor *in vivo*.

In conclusion, we identified and characterized the NKB/NK3 receptor system in spotted sea bass. We amplified spotted sea bass *tac3a* and *tac3b* cDNAs and determined their mRNA levels. We demonstrated that NKBs stimulated the mRNA levels of growth-related genes in the brain and brain-gut peptide (BGP)-related genes in the stomach and intestine. The present study is the first that demonstrates the involvement of the NKB/NK3 receptor system in the regulation of growth and gastrointestinal motility in spotted sea bass. Our data also provide valuable information for the further study of NKB/NK3 receptor preferential signaling pathways.

## Data Availability

All datasets generated for this study are included in the manuscript and/or the Supplementary Files.

## Ethics Statement

All spotted sea bass work was approved and performed in accordance with the respective Animal Research and Ethics Committees of Ocean University of China.

## Author Contributions

XQ, HW, and YLi designed the study. ZZ performed the RNA extraction and cDNA preparation. YLiu and LL performed in sequence analysis, tissue expression analysis. ZZ and QL performed the *in situ* hybirdization (ISH). ZZ and LW performed cell culture and tissue incubation experiment. WL and YZ performed samples collection. ZZ wrote the manuscript. XQ provided manuscript editing and feedback. All authors read and approved the final manuscript.

### Conflict of Interest Statement

The authors declare that the research was conducted in the absence of any commercial or financial relationships that could be construed as a potential conflict of interest.
