# Supplementary material for: TAC3 Gene Products Regulate Brain and Digestive System Gene Expression in the Spotted Sea Bass (Lateolabrax maculatus)
Source: Front Endocrinol (Lausanne). 2019 Aug 14;10:556. doi: 10.3389/fendo.2019.00556 (PMC6702303; doi:10.3389/fendo.2019.00556)
Supplement: Supplementary file 1 [file Data_Sheet_1.docx]

Supplementary Material

## Supplementary Figures


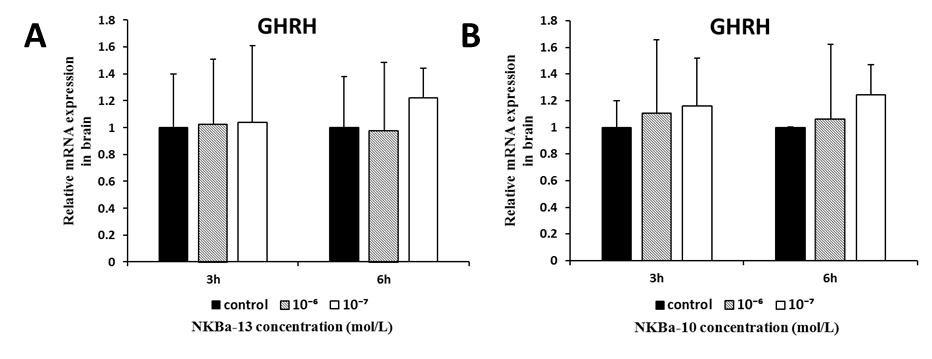


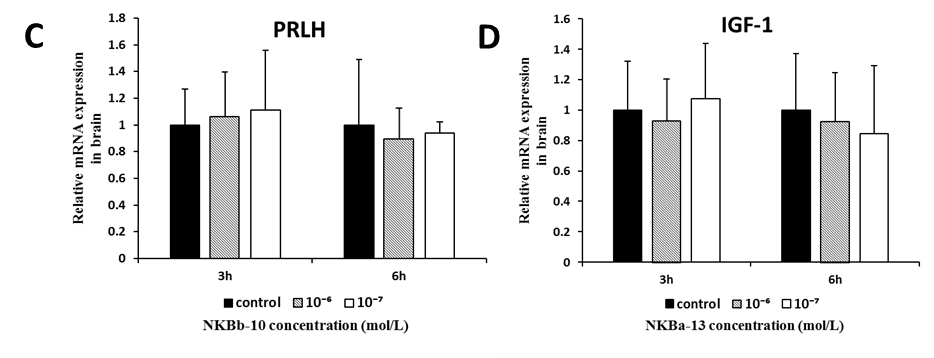


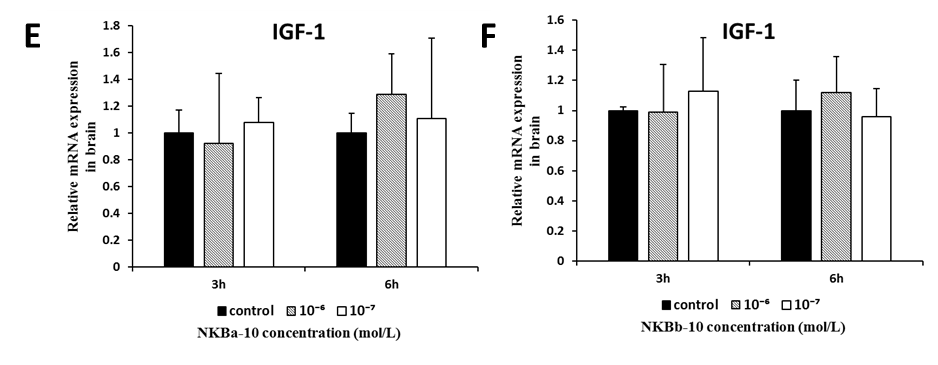


**
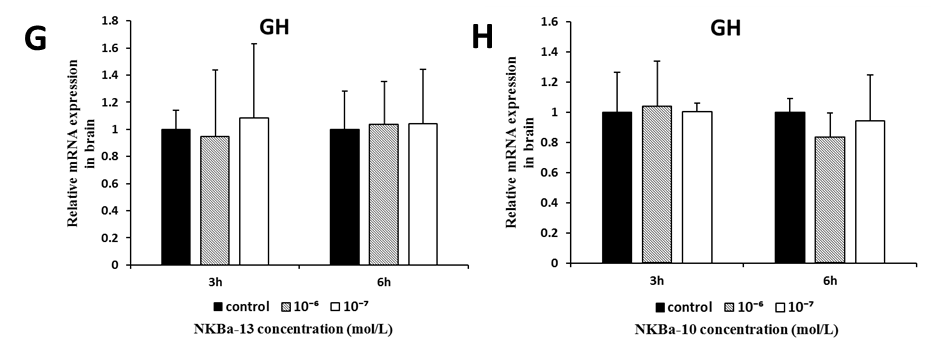
**

**
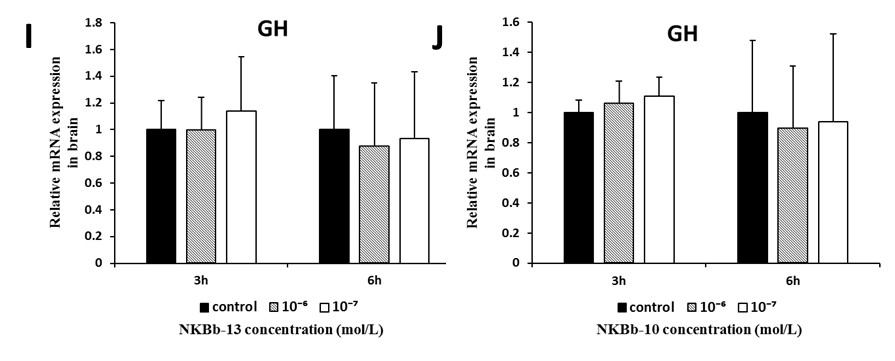
**

**Supplementary Figure 1.** Effect of NKBs on the expression of growth-related genes in the brain. **(A-B)** Effect of NKBa-13 and NKBa-10 on the mRNA level of GHRH in the brain; **(C)** Effect of NKBb-10 on the mRNA level of PRLH brain; **(D-F)** Effect of NKBa-13, NKBa-10and NKBb-10 on the mRNA level of IGF-1 brain; **(G-J)** Effect of NKBA-13, NKBa-10, NKBb-13and NKBb-10 on the mRNA level of GH brain; Significant differences were noted by different letters in each concentrations (*P*＜0.05)..


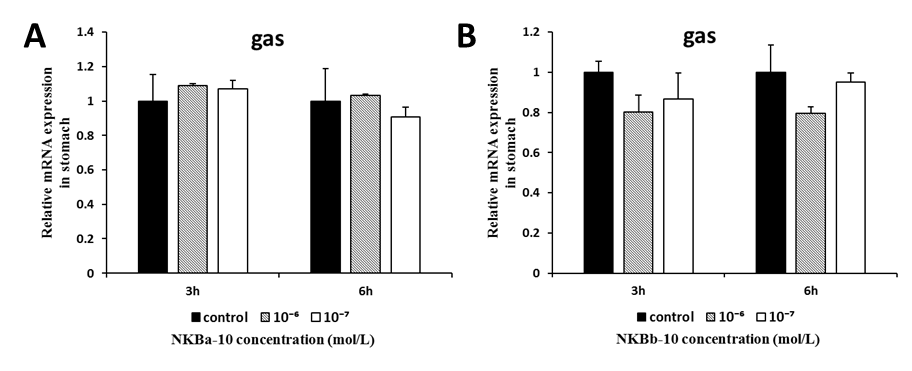


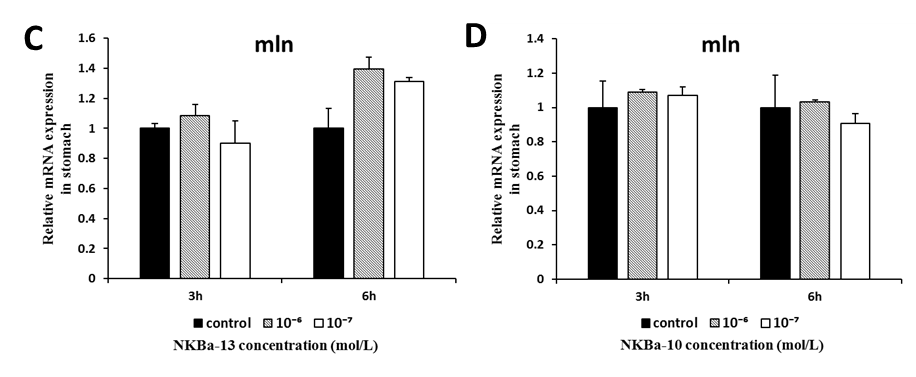


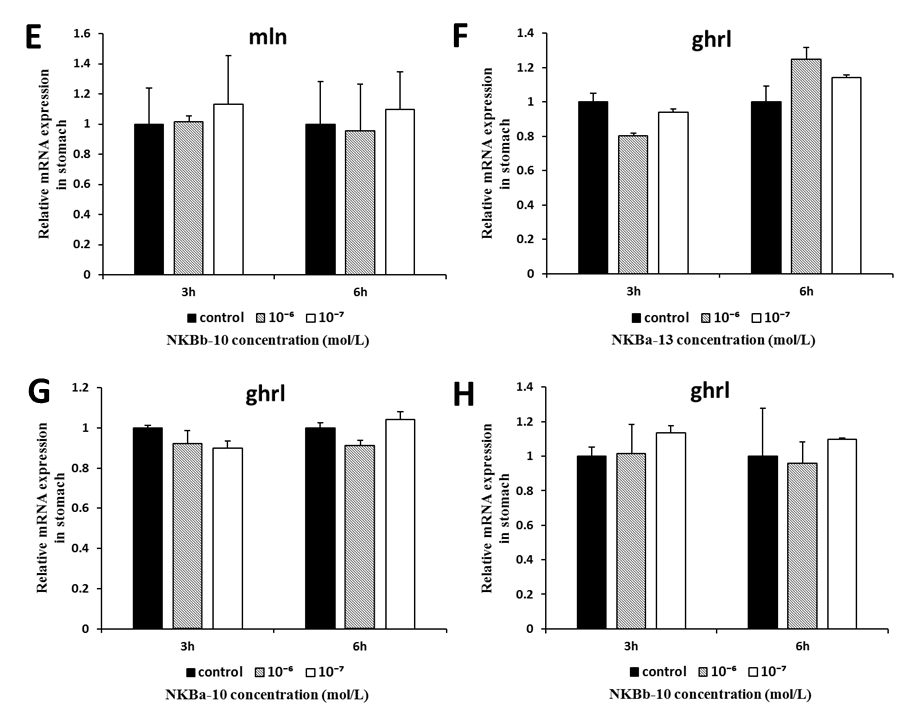


**Supplementary Figure 2.** Effect of NKBs on the expression of brain-gut peptide (BGP)-related genes in stomach. **(A-B)** Effect of NKBa-10 and NKBb-10 on the mRNA level of *gas* in stomach; **(C-E)** Effect of NKBa-13, NKBa-10, NKBb-10 on the mRNA level of *mln* and *ghrl* in stomach; Significant differences were noted by different letters in each concentrations (*P*＜0.05).

**
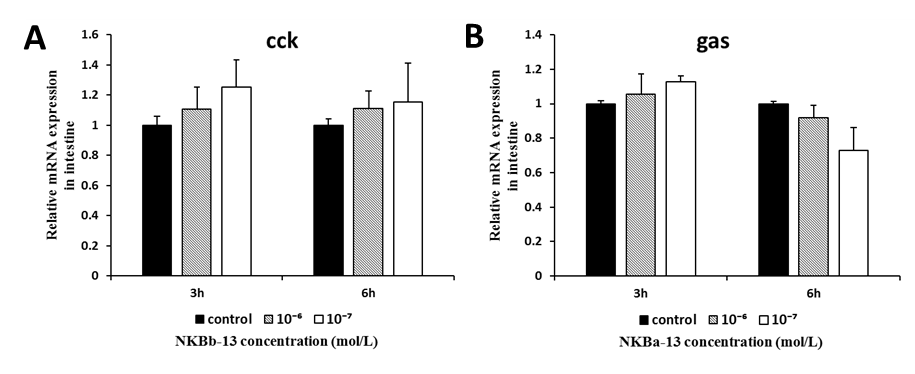
**

**
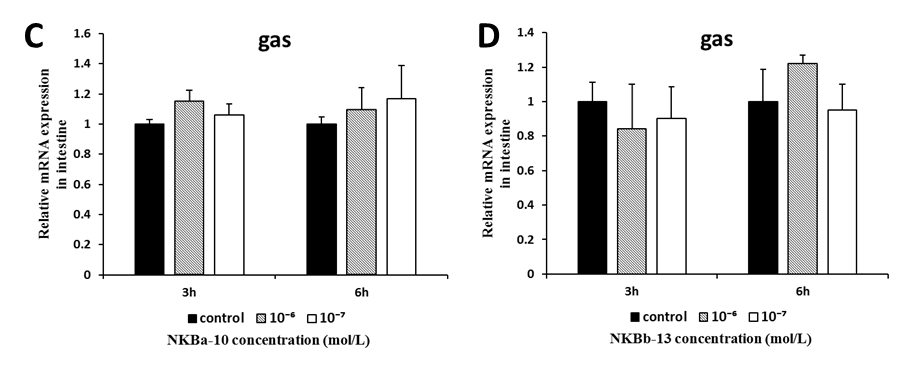
**

**
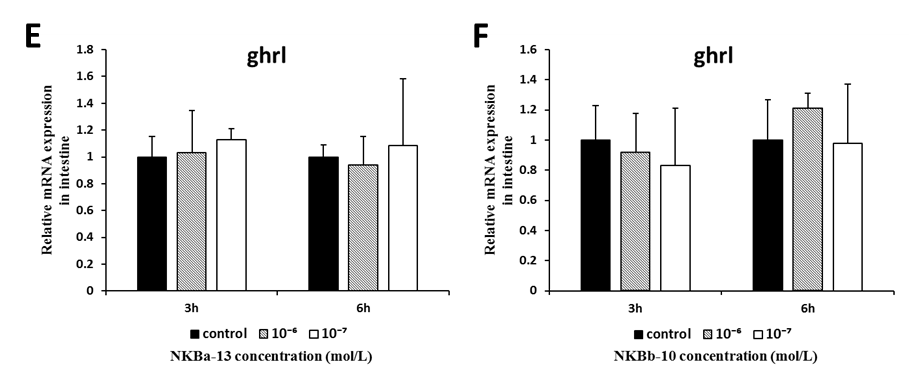
**

**
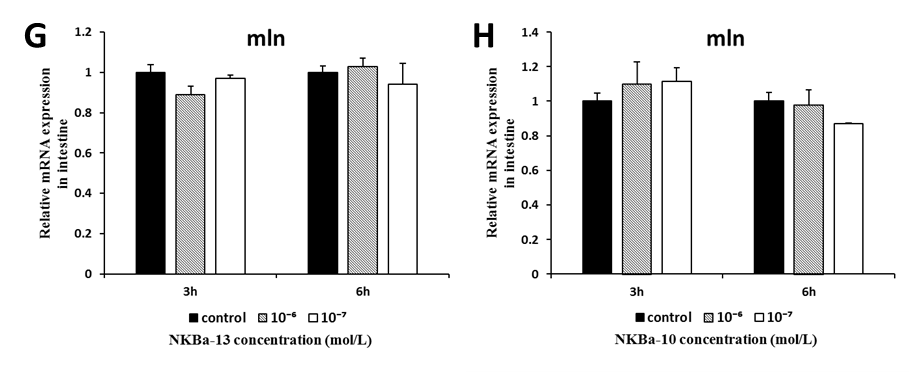
**

**
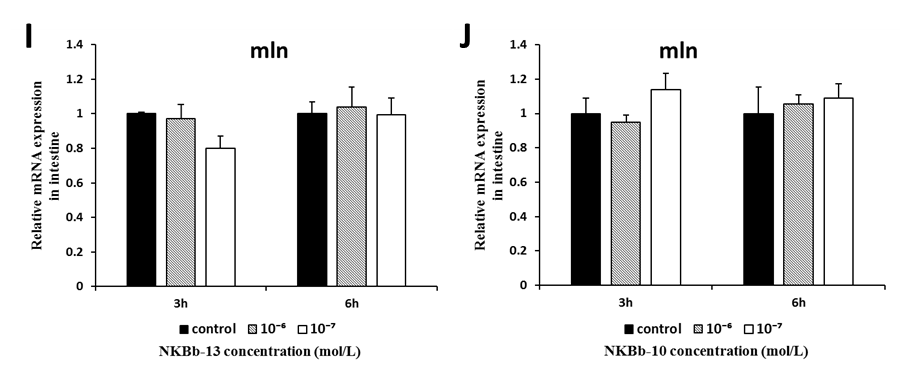
**

**Supplementary Figure 3.** Effect of NKBs on the expression of brain-gut peptide (BGP)-related genes in intestine. **(A)** Effect of NKBb-13 on the mRNA level of *cck* in intestine; **(B-D)** Effect of NKBa-13, NKBa-10, NKBb-13on the mRNA level of *gas* in intestine; **(E-F)** Effect of NKBa-13, NKBb-10 on the mRNA level of *ghrl* in intestine; **(G-J)** Effect of NKBa-13, NKBa-10, NKBb-13 and NKBb-10 on the mRNA level of *mln* in intestine. Significant differences were noted by different letters in each concentrations (*P*＜0.05).


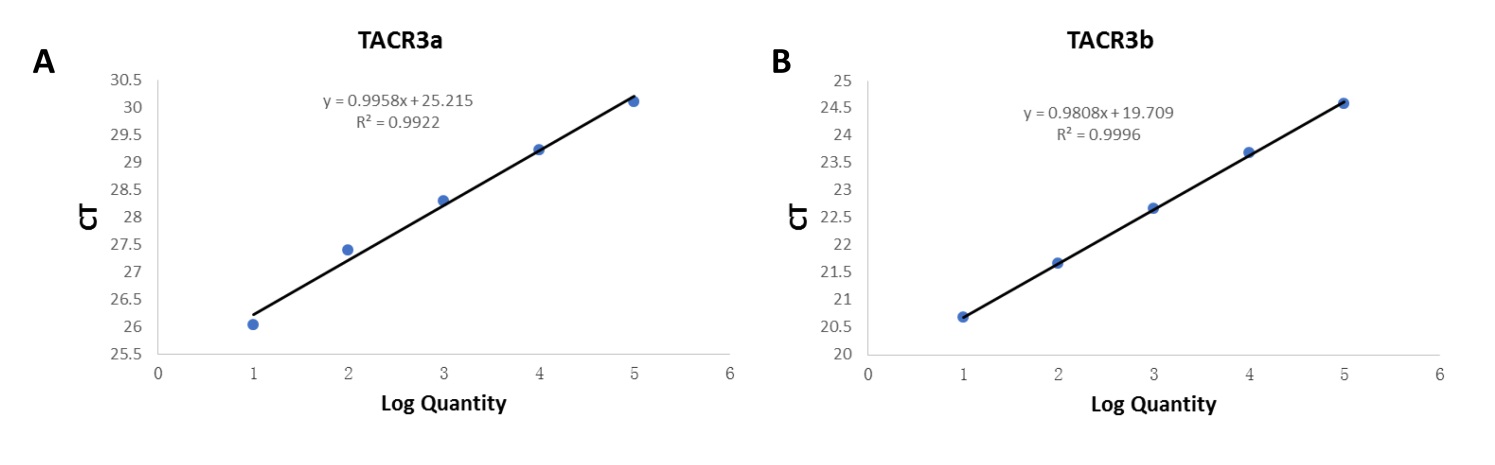


**Supplementary Figure 3.** Standard dilution curves of the TAC3R qPCR. **(A)** Standard dilution curves of the *tacr3a* qPCR. PCR efficiency is 100%. **(B)** Standard dilution curves of the *tacr3b* qPCR. PCR efficiency is 102%.


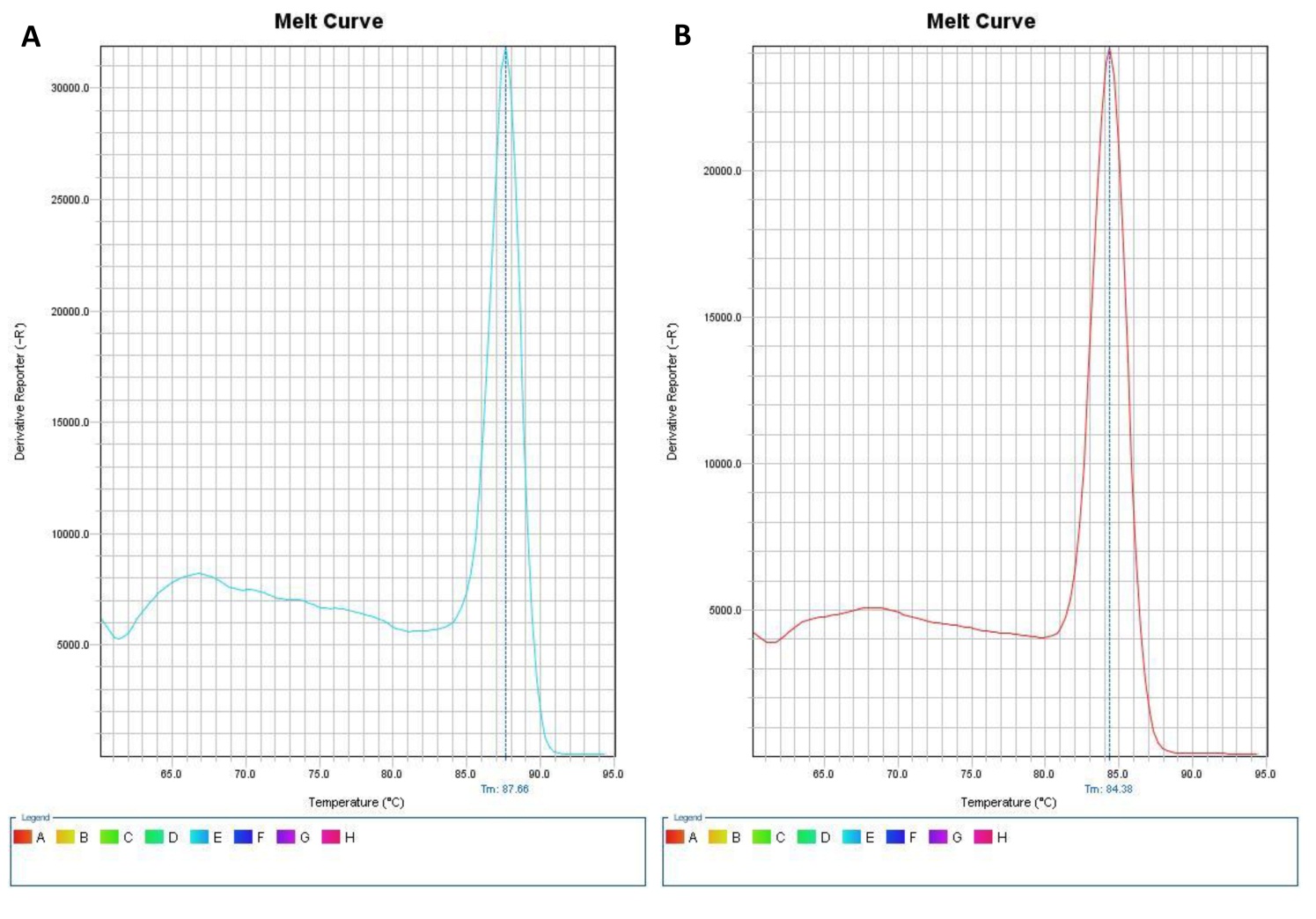


**Supplementary Figure 4.** Melt curves of the *tacr3s* qPCR. **(A)** Melt curve of the *tacr3a* qPCR. **(B)** Melt curve of the *tacr3b* qPCR.

## Supplementary sequencing results of *tac3s* and *tacr3s* for qRCR

**Tac3a:**

GAGGAGAGGATTGCTGTTGGTGACTCTGTTCCTCATCATGAAACTTCGATACAGCCAG

TCCAGATGTGAGGAGCCAGGATCGCGCAGATCATCCTCAGATCCGACCGTAGGCTTGGA

**Tac3b:**

TTGCTGCGGAACACAAGGATGGCTTGTCCGTGTACCGAGGAGTATTTACAACCAAGGAGAGGCCGACTCATCAATCATGGCAGGCAGAGGTT

**Tacr3a:**

AGCTACGACGAGTTGGAACTACGCTCTACACGGCTGCATCCAACACGCCAGAGCAGCATGTACACACTATCACCAATGGATACCAGCATGGTGGTGGTTTACGACCCTGCCGAGGGCGATGGCGGTGCTGGCGGTGGCTCTGGGGGAAAGCATTCCCTGTCCTCTCGGAAGAGAAGCTACATCACATCTCGCCACACGG

**Tacr3b:**

CAAGCGGATGCGGACTGTCACCAACTATTGCCTGCTCAACCTGGCCTTCTCCGACGTGTCGATGGCGGCGTTCAACACGCTCATCAACTTCATCTACGCGGCGCACGGAGAGTGGTACTTTGGAGAGGTGTACTGCAGGTTCCACAACTTCTTCCCCGTCACCGCGGTGTTCGCCAGCATCTACTCCATGAC

## Supplementary Tables

# Table 1 The exact p values of NKBb-13’s treatment on *ghrh* mRNA epression in the brain

|  | 3h(control) | 3h(10⁻⁶M) | 3h(10⁻⁷) | 6h(control) | 6h(10⁻⁶M) | 6h(10⁻⁷) |
| --- | --- | --- | --- | --- | --- | --- |
| 3h(control) |  |  |  |  |  |  |
| 3h(10⁻⁶M) | .967 |  |  |  |  |  |
| 3h(10⁻⁷) | .529 | .503 |  |  |  |  |
| 6h(control) | 1.000 | .967 | .529 |  |  |  |
| 6h(10⁻⁶M) | .005 | .003 | .010 | .003 |  |  |
| 6h(10⁻⁷) | .083 | .067 | .211 | .072 | .009 |  |

Table 2 The exact p values of NKBb-10’s treatment on *ghrh* mRNA epression in the brain

|  | 3h(control) | 3h(10⁻⁶M) | 3h(10⁻⁷) | 6h(control) | 6h(10⁻⁶M) | 6h(10⁻⁷) |
| --- | --- | --- | --- | --- | --- | --- |
| 3h(control) |  |  |  |  |  |  |
| 3h(10⁻⁶M) | .500 |  |  |  |  |  |
| 3h(10⁻⁷) | .490 | .987 |  |  |  |  |
| 6h(control) | 1.000 | .988 | .975 |  |  |  |
| 6h(10⁻⁶M) | .006 | .030 | .009 | .016 |  |  |
| 6h(10⁻⁷) | .521 | .973 | .960 | .985 | .020 |  |

Table 3 The exact p values of NKBa-13’s treatment on *prlh* mRNA epression in the brain

|  | 3h(control) | 3h(10⁻⁶M) | 3h(10⁻⁷) | 6h(control) | 6h(10⁻⁶M) | 6h(10⁻⁷) |
| --- | --- | --- | --- | --- | --- | --- |
| 3h(control) |  |  |  |  |  |  |
| 3h(10⁻⁶M) | .366 |  |  |  |  |  |
| 3h(10⁻⁷) | .685 | .200 |  |  |  |  |
| 6h(control) | 1.000 | .789 | .301 |  |  |  |
| 6h(10⁻⁶M) | .039 | .026 | .008 | .028 |  |  |
| 6h(10⁻⁷) | .035 | .019 | .046 | .036 | .757 |  |

Table 4 The exact p values of NKBa-10’s treatment on *prlh* mRNA epression in the brain

|  | 3h(control) | 3h(10⁻⁶M) | 3h(10⁻⁷) | 6h(control) | 6h(10⁻⁶M) | 6h(10⁻⁷) |
| --- | --- | --- | --- | --- | --- | --- |
| 3h(control) |  |  |  |  |  |  |
| 3h(10⁻⁶M) | .994 |  |  |  |  |  |
| 3h(10⁻⁷) | .373 | .369 |  |  |  |  |
| 6h(control) | 1.000 | .404 | .097 |  |  |  |
| 6h(10⁻⁶M) | .001 | .025 | .009 | .015 |  |  |
| 6h(10⁻⁷) | .046 | .309 | .039 | .047 | .023 |  |

Table 5 The exact p values of NKBb-13’s treatment on *prlh* mRNA epression in the brain

|  | 3h(control) | 3h(10⁻⁶M) | 3h(10⁻⁷) | 6h(control) | 6h(10⁻⁶M) | 6h(10⁻⁷) |
| --- | --- | --- | --- | --- | --- | --- |
| 3h(control) |  |  |  |  |  |  |
| 3h(10⁻⁶M) | .004 |  |  |  |  |  |
| 3h(10⁻⁷) | .719 | .007 |  |  |  |  |
| 6h(control) | 1.000 | .164 | .102 |  |  |  |
| 6h(10⁻⁶M) | .000 | .024 | .000 | .002 |  |  |
| 6h(10⁻⁷) | .000 | .058 | .000 | .001 | .032 |  |

Table 6 The exact p values of NKBb-13’s treatment on *igf1* mRNA epression in the brain

|  | 3h(control) | 3h(10⁻⁶M) | 3h(10⁻⁷) | 6h(control) | 6h(10⁻⁶M) | 6h(10⁻⁷) |
| --- | --- | --- | --- | --- | --- | --- |
| 3h(control) |  |  |  |  |  |  |
| 3h(10⁻⁶M) | 0.038 |  |  |  |  |  |
| 3h(10⁻⁷) | .440 | .048 |  |  |  |  |
| 6h(control) | 1.000 | .031 | .131 |  |  |  |
| 6h(10⁻⁶M) | .026 | .325 | .024 | .012 |  |  |
| 6h(10⁻⁷) | .278 | .043 | .741 | .074 | .018 |  |

Table 7 The exact p values of NKBa-13’s treatment on *gas* mRNA epression in the stomach

|  | 3h(control) | 3h(10⁻⁶M) | 3h(10⁻⁷) | 6h(control) | 6h(10⁻⁶M) | 6h(10⁻⁷) |
| --- | --- | --- | --- | --- | --- | --- |
| 3h(control) |  |  |  |  |  |  |
| 3h(10⁻⁶M) | .717 |  |  |  |  |  |
| 3h(10⁻⁷) | .602 | .872 |  |  |  |  |
| 6h(control) | 1.000 | .235 | .182 |  |  |  |
| 6h(10⁻⁶M) | .033 | .026 | .435 | .023 |  |  |
| 6h(10⁻⁷) | .051 | .098 | .129 | .060 | .036 |  |

Table 8 The exact p values of NKBb-13’s treatment on *gas* mRNA epression in the stomach

|  | 3h(control) | 3h(10⁻⁶M) | 3h(10⁻⁷) | 6h(control) | 6h(10⁻⁶M) | 6h(10⁻⁷) |
| --- | --- | --- | --- | --- | --- | --- |
| 3h(control) |  |  |  |  |  |  |
| 3h(10⁻⁶M) | .026 |  |  |  |  |  |
| 3h(10⁻⁷) | .056 | .385 |  |  |  |  |
| 6h(control) | .1.000 | .042 | .211 |  |  |  |
| 6h(10⁻⁶M) | .008 | .042 | .004 | .004 |  |  |
| 6h(10⁻⁷) | .040 | .073 | .031 | .016 | .788 |  |

Table 9 The exact p values of NKBb-13’s treatment on *mln* mRNA epression in the stomach

|  | 3h(control) | 3h(10⁻⁶M) | 3h(10⁻⁷) | 6h(control) | 6h(10⁻⁶M) | 6h(10⁻⁷) |
| --- | --- | --- | --- | --- | --- | --- |
| 3h(control) |  |  |  |  |  |  |
| 3h(10⁻⁶M) | .877 |  |  |  |  |  |
| 3h(10⁻⁷) | .996 | .872 |  |  |  |  |
| 6h(control) | 1.000 | .112 | .146 |  |  |  |
| 6h(10⁻⁶M) | .001 | .013 | .027 | .007 |  |  |
| 6h(10⁻⁷) | .805 | .926 | .800 | .095 | .043 |  |

Table 10 The exact p values of NKBb-13’s treatment on *ghrl* mRNA epression in the stomach

|  | 3h(control) | 3h(10⁻⁶M) | 3h(10⁻⁷) | 6h(control) | 6h(10⁻⁶M) | 6h(10⁻⁷) |
| --- | --- | --- | --- | --- | --- | --- |
| 3h(control) |  |  |  |  |  |  |
| 3h(10⁻⁶M) | .000 |  |  |  |  |  |
| 3h(10⁻⁷) | .000 | .000 |  |  |  |  |
| 6h(control) | 1.000 | .000 | .000 |  |  |  |
| 6h(10⁻⁶M) | .000 | .000 | .311 | .000 |  |  |
| 6h(10⁻⁷) | .003 | .000 | .042 | .000 | .016 |  |

Table 11 The exact p values of NKBa-13’s treatment on *cck* mRNA epression in the intestine

|  | 3h(control) | 3h(10⁻⁶M) | 3h(10⁻⁷) | 6h(control) | 6h(10⁻⁶M) | 6h(10⁻⁷) |
| --- | --- | --- | --- | --- | --- | --- |
| 3h(control) |  |  |  |  |  |  |
| 3h(10⁻⁶M) | .045 |  |  |  |  |  |
| 3h(10⁻⁷) | .051 | .948 |  |  |  |  |
| 6h(control) | 1.000 | .020 | .022 |  |  |  |
| 6h(10⁻⁶M) | .000 | .000 | .000 | .000 |  |  |
| 6h(10⁻⁷) | .032 | .020 | .069 | .014 | .000 |  |

Table 12 The exact p values of NKBa-10’s treatment on *cck* mRNA epression in the intestine

|  | 3h(control) | 3h(10⁻⁶M) | 3h(10⁻⁷) | 6h(control) | 6h(10⁻⁶M) | 6h(10⁻⁷) |
| --- | --- | --- | --- | --- | --- | --- |
| 3h(control) |  |  |  |  |  |  |
| 3h(10⁻⁶M) | .036 |  |  |  |  |  |
| 3h(10⁻⁷) | .839 | .043 |  |  |  |  |
| 6h(control) | 1.000 | .093 | .003 |  |  |  |
| 6h(10⁻⁶M) | .767 | .062 | .926 | .003 |  |  |
| 6h(10⁻⁷) | .003 | .039 | .074 | .020 | .205 |  |

Table 13 The exact p values of NKBb-10’s treatment on *cck* mRNA epression in the intestine

|  | 3h(control) | 3h(10⁻⁶M) | 3h(10⁻⁷) | 6h(control) | 6h(10⁻⁶M) | 6h(10⁻⁷) |
| --- | --- | --- | --- | --- | --- | --- |
| 3h(control) |  |  |  |  |  |  |
| 3h(10⁻⁶M) | .161 |  |  |  |  |  |
| 3h(10⁻⁷) | .149 | .230 |  |  |  |  |
| 6h(control) | 1.000 | .187 | .950 |  |  |  |
| 6h(10⁻⁶M) | .001 | .000 | .000 | .000 |  |  |
| 6h(10⁻⁷) | .009 | .033 | .000 | .000 | .002 |  |

Table 14 The exact p values of NKBb-10’s treatment on *gas* mRNA epression in the intestine

|  | 3h(control) | 3h(10⁻⁶M) | 3h(10⁻⁷) | 6h(control) | 6h(10⁻⁶M) | 6h(10⁻⁷) |
| --- | --- | --- | --- | --- | --- | --- |
| 3h(control) |  |  |  |  |  |  |
| 3h(10⁻⁶M) | .058 |  |  |  |  |  |
| 3h(10⁻⁷) | .237 | .358 |  |  |  |  |
| 6h(control) | 1.000 | .161 | .070 |  |  |  |
| 6h(10⁻⁶M) | .005 | .000 | .001 | .000 |  |  |
| 6h(10⁻⁷) | .023 | .038 | .040 | .037 | .009 |  |

Table 15 The exact p values of NKBa-10’s treatment on *ghrl* mRNA epression in the intestine

|  | 3h(control) | 3h(10⁻⁶M) | 3h(10⁻⁷) | 6h(control) | 6h(10⁻⁶M) | 6h(10⁻⁷) |
| --- | --- | --- | --- | --- | --- | --- |
| 3h(control) |  |  |  |  |  |  |
| 3h(10⁻⁶M) | .143 |  |  |  |  |  |
| 3h(10⁻⁷) | .093 | .802 |  |  |  |  |
| 6h(control) | 1.000 | .851 | .950 |  |  |  |
| 6h(10⁻⁶M) | .008 | .005 | .009 | .010 |  |  |
| 6h(10⁻⁷) | .027 | .044 | .047 | .031 | .013 |  |

Table 16 The exact p values for tissue expression analysis results of the *tac3a* mRNA levels.

| TAC3a | telencephalon | hypothalamus | midbrain | cerebellum | medulla oblongata | pituitary | kidney | muscle | gill | liver | Head kidney | spleen | heart | testis | stomach | intestine |
| --- | --- | --- | --- | --- | --- | --- | --- | --- | --- | --- | --- | --- | --- | --- | --- | --- |
| elencephalon |  |  |  |  |  |  |  |  |  |  |  |  |  |  |  |  |
| hypothalamus | .000 |  |  |  |  |  |  |  |  |  |  |  |  |  |  |  |
| midbrain | .021 | .000 |  |  |  |  |  |  |  |  |  |  |  |  |  |  |
| cerebellum | .612 | .000 | .006 |  |  |  |  |  |  |  |  |  |  |  |  |  |
| medulla oblongata | .000 | .000 | .018 | .000 |  |  |  |  |  |  |  |  |  |  |  |  |
| pituitary | .469 | .000 | .003 | .826 | .000 |  |  |  |  |  |  |  |  |  |  |  |
| kidney | .565 | .000 | .005 | .945 | .000 | .881 |  |  |  |  |  |  |  |  |  |  |
| muscle | .544 | .000 | .013 | .847 | .000 | .989 | .891 |  |  |  |  |  |  |  |  |  |
| gill | .747 | .000 | .009 | .854 | .000 | .686 | .799 | .731 |  |  |  |  |  |  |  |  |
| liver | .461 | .000 | .003 | .817 | .000 | .990 | .871 | .997 | .677 |  |  |  |  |  |  |  |
| Head kidney | .466 | .000 | .003 | .823 | .000 | .997 | .878 | .991 | .684 | .993 |  |  |  |  |  |  |
| spleen | .462 | .000 | .003 | .818 | .000 | .991 | .872 | .996 | .678 | .999 | .994 |  |  |  |  |  |
| heart | .516 | .000 | .004 | .885 | .000 | .940 | .940 | .940 | .742 | .930 | .937 | .931 |  |  |  |  |
| testis | .021 | .000 | .996 | .006 | .018 | .003 | .005 | .014 | .009 | .003 | .003 | .003 | .004 |  |  |  |
| stomach | .464 | .000 | .003 | .820 | .000 | .993 | .874 | .994 | .680 | .997 | .996 | .998 | .933 | .003 |  |  |
| intestine | .000 | .000 | .001 | .000 | .314 | .000 | .000 | .000 | .000 | .000 | .000 | .000 | .000 | .001 | .000 |  |

Table 17 The exact p values for tissue expression analysis results of the *tac3b* mRNA levels.

| TAC3b | telencephalon | hypothalamus | midbrain | cerebellum | medulla oblongata | pituitary | kidney | muscle | gill | liver | Head kidney | spleen | heart | testis | stomach | intestine |
| --- | --- | --- | --- | --- | --- | --- | --- | --- | --- | --- | --- | --- | --- | --- | --- | --- |
| elencephalon |  |  |  |  |  |  |  |  |  |  |  |  |  |  |  |  |
| hypothalamus | .000 |  |  |  |  |  |  |  |  |  |  |  |  |  |  |  |
| midbrain | .000 | .964 |  |  |  |  |  |  |  |  |  |  |  |  |  |  |
| cerebellum | .000 | .967 | .997 |  |  |  |  |  |  |  |  |  |  |  |  |  |
| medulla oblongata | .000 | .645 | .613 | .616 |  |  |  |  |  |  |  |  |  |  |  |  |
| pituitary | .000 | .006 | .005 | .005 | .019 |  |  |  |  |  |  |  |  |  |  |  |
| kidney | .000 | .945 | .910 | .913 | .695 | .007 |  |  |  |  |  |  |  |  |  |  |
| muscle | .000 | .954 | .990 | .987 | .604 | .005 | .900 |  |  |  |  |  |  |  |  |  |
| gill | .000 | .967 | .931 | .934 | .675 | .006 | .979 | .921 |  |  |  |  |  |  |  |  |
| liver | .000 | .934 | .970 | .967 | .587 | .005 | .880 | .980 | .901 |  |  |  |  |  |  |  |
| Head kidney | .000 | .930 | .895 | .898 | .709 | .007 | .985 | .885 | .964 | .865 |  |  |  |  |  |  |
| spleen | .000 | .975 | .990 | .992 | .622 | .005 | .920 | .979 | .941 | .959 | .905 |  |  |  |  |  |
| heart | .000 | .977 | .941 | .944 | .666 | .006 | .968 | .931 | .990 | .911 | .954 | .952 |  |  |  |  |
| testis | .000 | .000 | .000 | .000 | .000 | .000 | .000 | .000 | .000 | .000 | .000 | .000 | .000 |  |  |  |
| stomach | .000 | .000 | .000 | .000 | .000 | .040 | .000 | .000 | .000 | .000 | .000 | .000 | .000 | .074 |  |  |
| intestine | .001 | .000 | .000 | .000 | .000 | .000 | .000 | .000 | .000 | .000 | .000 | .000 | .000 | .001 | .000 |  |

Table 18 The exact p values for tissue expression analysis results of the *tacr3a* mRNA levels.

| TACR3a | telencephalon | hypothalamus | midbrain | cerebellum | medulla oblongata | pituitary | kidney | muscle | gill | liver | Head kidney | heart | testis | stomach | intestine |
| --- | --- | --- | --- | --- | --- | --- | --- | --- | --- | --- | --- | --- | --- | --- | --- |
| elencephalon |  |  |  |  |  |  |  |  |  |  |  |  |  |  |  |
| hypothalamus | .709 |  |  |  |  |  |  |  |  |  |  |  |  |  |  |
| midbrain | .566 | .346 |  |  |  |  |  |  |  |  |  |  |  |  |  |
| cerebellum | .407 | .647 | .165 |  |  |  |  |  |  |  |  |  |  |  |  |
| medulla oblongata | .560 | .854 | .235 | .760 |  |  |  |  |  |  |  |  |  |  |  |
| pituitary | .047 | .101 | .012 | .230 | .115 |  |  |  |  |  |  |  |  |  |  |
| kidney | .000 | .000 | .000 | .000 | .000 | .000 |  |  |  |  |  |  |  |  |  |
| muscle | .722 | .986 | .354 | .635 | .839 | .098 | .000 |  |  |  |  |  |  |  |  |
| gill | .000 | .000 | .000 | .000 | .000 | .000 | .000 | .000 |  |  |  |  |  |  |  |
| liver | .022 | .051 | .005 | .127 | .056 | .736 | .000 | .049 | .000 |  |  |  |  |  |  |
| Head kidney | .093 | .042 | .258 | .015 | .020 | .001 | .000 | .044 | .000 | .000 |  |  |  |  |  |
| heart | .000 | .000 | .002 | .000 | .000 | .000 | .000 | .000 | .004 | .000 | .040 |  |  |  |  |
| testis | .015 | .036 | .003 | .094 | .039 | .623 | .000 | .035 | .000 | .877 | .000 | .000 |  |  |  |
| stomach | .028 | .066 | .006 | .169 | .073 | .924 | .000 | .064 | .000 | .790 | .000 | .000 | .667 |  |  |
| intestine | .036 | .080 | .009 | .189 | .091 | .908 | .000 | .077 | .000 | .824 | .000 | .000 | .707 | .977 |  |

Table 19 The exact p values for tissue expression analysis results of the *tacr3b* mRNA levels.

| TACR3b | telencephalon | hypothalamus | midbrain | cerebellum | medulla oblongata | pituitary | kidney | muscle | gill | liver | Head kidney | heart | testis | stomach | intestine |
| --- | --- | --- | --- | --- | --- | --- | --- | --- | --- | --- | --- | --- | --- | --- | --- |
| elencephalon |  |  |  |  |  |  |  |  |  |  |  |  |  |  |  |
| hypothalamus | .076 |  |  |  |  |  |  |  |  |  |  |  |  |  |  |
| midbrain | .001 | .000 |  |  |  |  |  |  |  |  |  |  |  |  |  |
| cerebellum | .000 | .000 | .250 |  |  |  |  |  |  |  |  |  |  |  |  |
| medulla oblongata | .415 | .010 | .008 | .000 |  |  |  |  |  |  |  |  |  |  |  |
| pituitary | .000 | .000 | .562 | .562 | .002 |  |  |  |  |  |  |  |  |  |  |
| kidney | .508 | .016 | .006 | .000 | .877 | .001 |  |  |  |  |  |  |  |  |  |
| muscle | .974 | .052 | .001 | .000 | .402 | .000 | .499 |  |  |  |  |  |  |  |  |
| gill | .000 | .000 | .319 | .874 | .001 | .673 | .000 | .000 |  |  |  |  |  |  |  |
| liver | .002 | .000 | .631 | .091 | .017 | .274 | .011 | .001 | .127 |  |  |  |  |  |  |
| Head kidney | .000 | .000 | .436 | .704 | .001 | .841 | .001 | .000 | .824 | .193 |  |  |  |  |  |
| heart | .000 | .000 | .547 | .524 | .001 | .985 | .001 | .000 | .639 | .246 | .816 |  |  |  |  |
| testis | .000 | .000 | .763 | .392 | .004 | .780 | .003 | .000 | .484 | .423 | .632 | .779 |  |  |  |
| stomach | .000 | .000 | .049 | .438 | .000 | .167 | .000 | .000 | .346 | .010 | .240 | .132 | .096 |  |  |
| intestine | .002 | .000 | .834 | .409 | .010 | .757 | .007 | .001 | .493 | .519 | .625 | .756 | .952 | .125 |  |
